# Distinct CaMKII neurons in anterior cingulate cortex regulate anxiety-like behavior and appetite suppression in a model of inflammatory pain

**DOI:** 10.3389/fphar.2026.1897103

**Published:** 2026-09-03

**Authors:** Jiandong Yu, Huiyue Han, Yuehan You, Bingjing Sun, Xinyi Wang, Jing Guan, Menghan Gu, Jing Feng, Zinan Zheng, Rongjing Ge, Li Mei

**Affiliations:** 1 Department of Neurosurgery, the First Affiliated Hospital of Bengbu Medical University, Bengbu, Anhui, China; 2 Laboratory of Brain and Psychiatric Disease, Bengbu Medical University, Bengbu, Anhui, China; 3 Anhui Engineering Research Center for Neural Regeneration Technology and Medical New Materials, Bengbu Medical University, Bengbu, Anhui, China; 4 Department of Pathophysiology, Bengbu Medical University, Bengbu, Anhui, China; 5 Department of Anesthesiology, Guangdong Provincial People’s Hospital, Guangdong Academy of Medical Sciences, Southern Medical University, Guangzhou, Guangdong, China; 6 Longhu Laboratory of Bengbu Medical University, Bengbu, Anhui, China

**Keywords:** anterior cingulate cortex, basolateral amygdala, cholera toxin subunit b, chronic pain, nucleus accumbens

## Abstract

**Background:**

Chronic Inflammatory pain can lead to emotional abnormalities such as anxiety and a decreased appetite. However, the specific mechanisms underlying these abnormalities remain unclear. The anterior cingulate cortex (ACC), which plays a crucial role in integrating nociceptive perception and emotional responses to chronic inflammatory pain, is an ideal region for studying how pain influences anxiety and appetite.

**Methods:**

In this study, we employed a variety of techniques, including fiber photometry recordings, neuroanatomical tract tracing, optogenetics, pharmacogenetics, and behavioral experiments, to investigate the neuronal circuits in the ACC of mice with chronic inflammatory pain.

**Results:**

We identified two distinct CaMKII neurons in the ACC: one that projects to the basolateral amygdala (BLA) and is associated with anxiety-like behavior and another that projects to the nucleus accumbens (NAc) and regulates appetite suppression. Furthermore, we found that these two circuits are largely independent. And the intrinsic excitability and synaptic inputs in ACCC_aMKII_→BLA neurons and ACCC_aMKII_→NAc neurons are differentially influenced by chronic inflammatory pain.

**Conclusion:**

Our findings offer valuable insights into the circuit mechanisms in the ACC that regulate anxiety-like and appetite-suppression behaviors.

## Graphical Abstract

**Figure:**
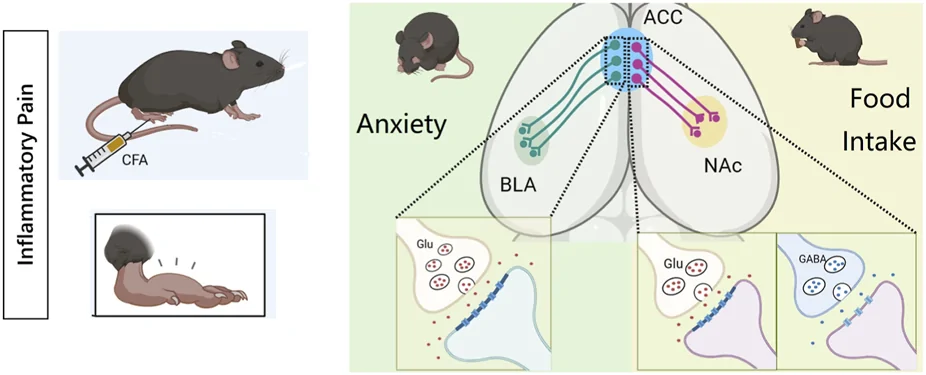

## Introduction

Chronic pain is a common disorder that significantly affects the quality of life for more than 20%–30% of the world’s population (Cohen et al., 2021; Gatchel et al., 2014), frequently driving negative emotional abnormalities, including anxiety and depression (Baliki et al., 2015; Eccleston et al., 2013). However, neuronal circuitry mechanisms underlying these comorbidities are not yet fully understood.

The anterior cingulate cortex (ACC), the frontal region of the cingulate cortex (or limbic lobe), plays a critical role in integrating nociceptive perception and emotional responses in chronic pain (Vogt, 2005; Xu et al., 2008; Koga et al., 2015). It receives nociceptive input from areas such as the thalamus, amygdala, and primary somatosensory cortex, and sends projections to the periaqueductal grey (PAG) for pain modulation and to limbic regions like the amygdala and nucleus accumbens (NAc) for emotional and motivational processing (Gabbott et al., 2005; Smith et al., 2021). The ACC cells are heterogeneous, and it remains unclear how specific downstream targets mediate the distinct components of pain (Smith et al., 2021; Gao et al., 2020).

Disordered eating is always associated with chronic pain (Potapenko et al., 2024; Sim et al., 2021). Feeding behavior, which is essential for survival, is regulated by numerous brain regions, including the nucleus accumbens (Lee et al., 2021). As a core basal ganglia hub, the NAc interconnects limbic and motor circuits, converting emotional states into motivated motor outputs. NAc is the center for processing reward and punishment (Phelps et al., 2014; Ruff and Ernst, 2014) and plays a crucial role in various psychiatric disorders, including addiction, depression, and substance abuse (De and d’Exaerde, 2021; Xu et al., 2020). Activation of ACC→NAc projections has been found to lead to positional avoidance, photic hypersensitivity and effortful decisions in mice (Smith et al., 2021; Gao et al., 2020; Fetcho et al., 2024; Ma et al., 2023). The functional connectivity based in the NAc reflects significant individual differences in appetite and body weight among patients with depression (Kroemer et al., 2022). However, whether the ACC→NAc pathway is involved in pain-related appetite suppression remains unclear.

Unpleasant experience of pain can lead to anxiety. There are several different brain areas such as the prefrontal cortex (Kenwood et al., 2022), hippocampus (Cerquetella et al., 2025; Engin et al., 2016), insular cortex (Potapenko et al., 2024; Labrakakis, 2023), and periaqueductal gray (Xie et al., 2023) are involved in both pain and anxiety (Chen et al., 2022; Zhuo, 2016). The basolateral amygdala (BLA) is the primary output region of the amygdala and is considered a crucial brain region involved in anxiety and fear memory (Janak and Tye, 2015; Nicolas et al., 2023; Ressler and Maren, 2019). A recent study by Gao reports that the prelimbic cortex to BLA circuit modulates anxiety-like behaviors caused by neuropathic pain (Gao et al., 2023), suggesting that the BLA may act as a pivotal node chronic pain-related anxiety. The ACC sends projections to the BLA and is involved in the generalized fear response in mice (Jhang et al., 2018; Ortiz et al., 2019). Furthermore, ACC is important for anxiety-like behavior and allodynia following chronic pancreatitis and early life maternal separation (Jiang et al., 2023; Ren et al., 2022). However, whether the ACC projection to BLA drives pain-induced anxiety-like behavior remains unclear (Koga et al., 2015).

Here, we analyzed the function of the ACC^CaMKII^→BLA and ACC^CaMKII^→NAc circuits in inflammatory pain. Using optogenetic, pharmacogenetic, and behavioral approaches, we demonstrated that two distinct CaMKII neuronal populations in the ACC project to the BLA and NAc, regulating anxiety-like behaviors and appetite suppression, respectively, in a mouse model of inflammatory pain. Electrophysiological recordings revealed that these populations show differential alterations under chronic pain. Our study collectively provides evidence revealing the circuit mechanisms underlying anxiety and decreased appetite in inflammatory pain.

## Methods

### Animals

In all of the experiments, male C57BL/6 mice (8–9 weeks, four per cage) were housed with a 12-h light/dark cycle (lights on at 8:00 a.m. and light off at 8:00 p.m.). All animals were given food and water under standard conditions (21 °C–26 °C).

### Stereotaxic injections and optic fiber implantation

For all injections, mice were anesthetized with pentobarbital (intraperitoneal, 75 mg/kg). Injections were performed using a microsyringe pump (Legato® 130 Syringe Pump, kdScientific) at a rate of 50 nl/min. The glass pipette was retracted after 10 min and then slowly withdrawn. Optical fibers (200 μm core diameter, 0.37 numerical aperture (NA)) implants for optogenetics experiments (AAV-CaMKII-hChR2-EYFP, Obio Technology, China) were implanted to the same coordinates as the viral injections, with a −0.3 mm variance. For optogenetic activation experiments, both control and ChR2 virus injected mice were stimulated using a 465 nm blue laser (20 Hz, 5 m, 2–5 mW). Referring to The Paxinos & Franklin Mouse Brain in Stereotaxic Coordinates (5th edition), the coordinates for ACC were set as AP: +0.8 mm; ML: −/+0.3 mm; DV: −1.5 mm, NAc as AP: +1.1 mm; ML: −/+1.20 mm; DV: −4.6 mm, BLA as AP: −1.6 mm; ML: −/+3.35 mm; DV: −4.9 mm.

For chemogenetic (AAV-CaMkIIa-hM4Di-mCherry, Obio Technology, AAV retro-hSyn-cre and AAV CaMKII-DIO-hM4Di-EGFP, BrainCase, China) modulation of neurons, mice were random intraperitoneally injected with CNO (5 mg/kg) or saline 0.5 h before behavior tests. All AAV vectors used for chemogenetic inhibition of ACC circuits were delivered via bilateral stereotaxic injections into the ACC, BLA, and NAc.

Only when the viral expression area completely covered the target range were the data of the mouse included for analysis.

### Fiber photometry recordings

Fiber photometry recordings were performed using multichannel fiber photometry recording system (R811/821, RWD). Mice were injected with 200 nL of the rAAV-CaMKIIα-GCaMP6m-WPRE-hGH polyA (AAV2/9, BrainVTA) into the right ACC at a rate of 20 nL/min. LED light at 470 nm was used to measure neuronal activity, while 410 nm light served as the autofluorescence background. The change in calcium transients was calculated using the formula ΔF/F = (F–F0)/F0, where F0 represents the signals from the 410 nm light and F represents the signals from the 470 nm light for each trial.

### Inflammatory pain model

Inflammatory pain was induced in mice using complete Freund’s adjuvant (CFA). The skin of the hindpaw planta was disinfected with 75% ethanol, and then 10 μl of CFA was injected subcutaneously into the left hind paw planta. The thickness of the left hind paw doubled the next day, indicating the successful establishment of the inflammatory pain model.

### von frey filament test

The mechanical withdrawal threshold was measured using manual (NC12775-99, North Coast, United States) or electronic (Zhongshi, Beijing, China) von Frey filaments on the middle of the plantar surface of the left hind paws. Mice were placed individually in plastic chambers on a wire mesh grid. A filament probe was inserted, and the pressure was increased gradually. A positive response was considered when a mouse withdrew or licked its paw.

### Open-field test (OFT)

Before testing, mice were habituated in the testing house for 1 h. Then each individual mouse was placed in the center of chamber (40 × 40 × 30 cm). Mice were gently placed and monitored for 5 min. The time mice spent in the center (15 × 15 cm square) and the total distance that mice explored in the open field were measured by ANY–maze (Stoelting, IL, United States).

### Elevated plus maze test (EPM)

The EPM was 50 cm above the floor. The maze consisted of two open arms (25 × 8 × 0.5 cm) and closed arms (25 × 8 × 12 cm) that extended from a central platform (8 × 8 cm). Each mouse was placed into the center facing a closed arm. A camera above the maze was used to record the movement of the mouse during a 5-min test.

### Feeding measurement

All acute feeding assays were conducted between 10:30 a.m. and 3:30 p.m. Prior to behavioral recording, all mice assigned to feeding tests underwent a five-day habituation protocol to reduce stress: animals were individually housed with food and water positioned at the upper-left cage corner for 3 h each day. Food consumption and body weight were quantified on the fifth habituation day (Tang et al., 2022).

For chemogenetic feeding experiments using DREADDs: DREADD-expressing mice were single-housed and received an intraperitoneal (i.p.) injection of clozapine-N-oxide (CNO, 5 mg/kg; Sigma) 0.5 h before food intake measurement. To account for non-specific off-target CNO activity at dopamine receptors (Jendryka et al., 2019), control animals received empty control AAV and an identical CNO dose. One-hour cumulative food intake was quantified by an experimenter blinded to experimental groups.

For optogenetic feeding measurements, each recording session spanned 3 h total. To mitigate behavioral disturbances caused by the head-mounted optical fiber, illumination was delivered in a block design: lights were off for the first hour, turned on for the second hour, and switched off again for the final hour of each feeding trial.

### Body weight measurement

Body weight was measured by a blind investigator during for 10 consecutive days. For food intake under chemogenetic modulation, saline was injected during day1-day3 and day7-day8. CNO was injected during day4-day6.

### Brain-slice electrophysiology

Slices were prepared as previously described (Yu et al., 2013). After being anesthetized with isoflurane, the mice were decapitated, and the brain was transferred into ice-cold artificial cerebrospinal fluid (ACSF, cutting solution) made of 2.5 mM KCl, 220 mM sucrose, 2.5 mM MgSO_4_, 1.3 mM CaCl_2_, 1 mM NaH_2_PO4, 26 mM NaHCO_3_, and 10 mM glucose. Coronal slices (300 µm) were cut using a vibrating microtome (Leica VT 1000S, Germany). The slices were then transferred to a chamber for a 20-min recovery at 32 °C and were subsequently kept at room temperature for an additional hour before recording. The chamber contained the recording ACSF (26 mM NaHCO_3_, 126 mM NaCl, 1.2 mM NaH_2_PO_4_, 3 mM KCl, 2 mM CaCl_2_, 1 mM MgSO_4_, and 10 mM glucose). All solutions were saturated with a 95% O_2_/5% CO_2_ (vol/vol) mixture. All action potential properties and excitability recordings were performed in the presence of NBQX (10 mM) and BMI (20 µM).

To record sEPSCs, pipettes filled with 30 mM KCl, 105 mM K-gluconate, 10 mM phosphocreatine, 10 mM HEPES, 4 mM ATP-Mg, 0.3 mM EGTA, 0.3 mM GTP-Na, and 5 mM QX-314 (pH 7.4, 290 mOsm). sEPSCs were recorded at a holding potential of −60 mV in the presence of BMI (20 µM). sIPSCs were recorded at a holding potential of −70 mV, with an internal solution containing 140 mM CsCl, 2 mM MgCl_2_, 1 mM CaCl_2_, 10 mM EGTA, 10 mM HEPES-CsOH, 2 mM ATP, and 5 mM QX-314. 1 mM kynurenic acid was added to the recording solution. For isolation of monosynaptic light-evoked responses, brain slices were bath-perfused with TTX plus 200 µM 4-AP. Co-administration of these two compounds blocks action potential-dependent terminal depolarization and polysynaptic transmission without disrupting ChR2-driven axon terminal depolarization or monosynaptic optical synaptic signals, consistent with prior work (Li et al., 2024).

Digidata1440 converter and Axoclamp-700 B amplifiers (Molecular Devices, Sunnyvale, CA, United States) were used. All analyses were performed using Clampfit 10.6 (Axon Instruments/Molecular Devices).

### c-fos immunohistochemistry and imaging

One hour after the von Frey filament test, mice were deeply anesthetized with 20% urethane and then perfused with ice-cold 0.9% saline and 4% paraformaldehyde. The brains were extracted, placed in 4% paraformaldehyde at 4 °C for 24 h, and subsequently incubated in sucrose gradient solutions (w/v, 20%, 30%) until they sank. Brain slices (20 µm) were incubated with 0.2% Triton X-100 for 8 min, blocked with 2% BSA and 10% normal donkey serum for 1 h. Subsequently, the slices were incubated overnight at 4 °C with Anti-c-fos antibody (Abcam, AB222699, 1:500), followed by incubation with the secondary antibody Alexa Fluor® 594 (Abcam, AB150080; 1:1,000) for 2 h. After rinsing, the slices were incubated in DAPI for 7 min. Finally, the slices were scanned and imaged with a confocal microscope (Olympus FV1200MPE) to visualize neurons.

### Reagents and drugs

The retrograde neuroanatomical tracer Alexa Fluor 488-conjugated cholera toxin subunit B (CTB) into the BLA and Alexa Fluor 555–CTB and all the chemical reagents were purchased from Sigma–Aldrich.

### Quantification and statistical analysis

Sample sizes, statistical tests used, and statistical significance are described in the figures and the figure Legends. Unless otherwise indicated, values are reported as mean ± SEM. Behavioral and quantification experiments were done in a blinded manner. One-way or one-way with repeated measures ANOVA statistical test was followed by *post hoc* comparisons. Mann-Whitney U test and t-test were used to compare two groups. In figures, asterisks show the obtained statistical significance *p < 0.05, **p < 0.01, ***p < 0.001. All statistical analysis was performed using GraphPad Prism 5.0 software (GraphPad).

## Result

### ACC modulates anxiety-like behavior and appetite suppression induced by inflammatory pain in mice

**FIGURE 1:**
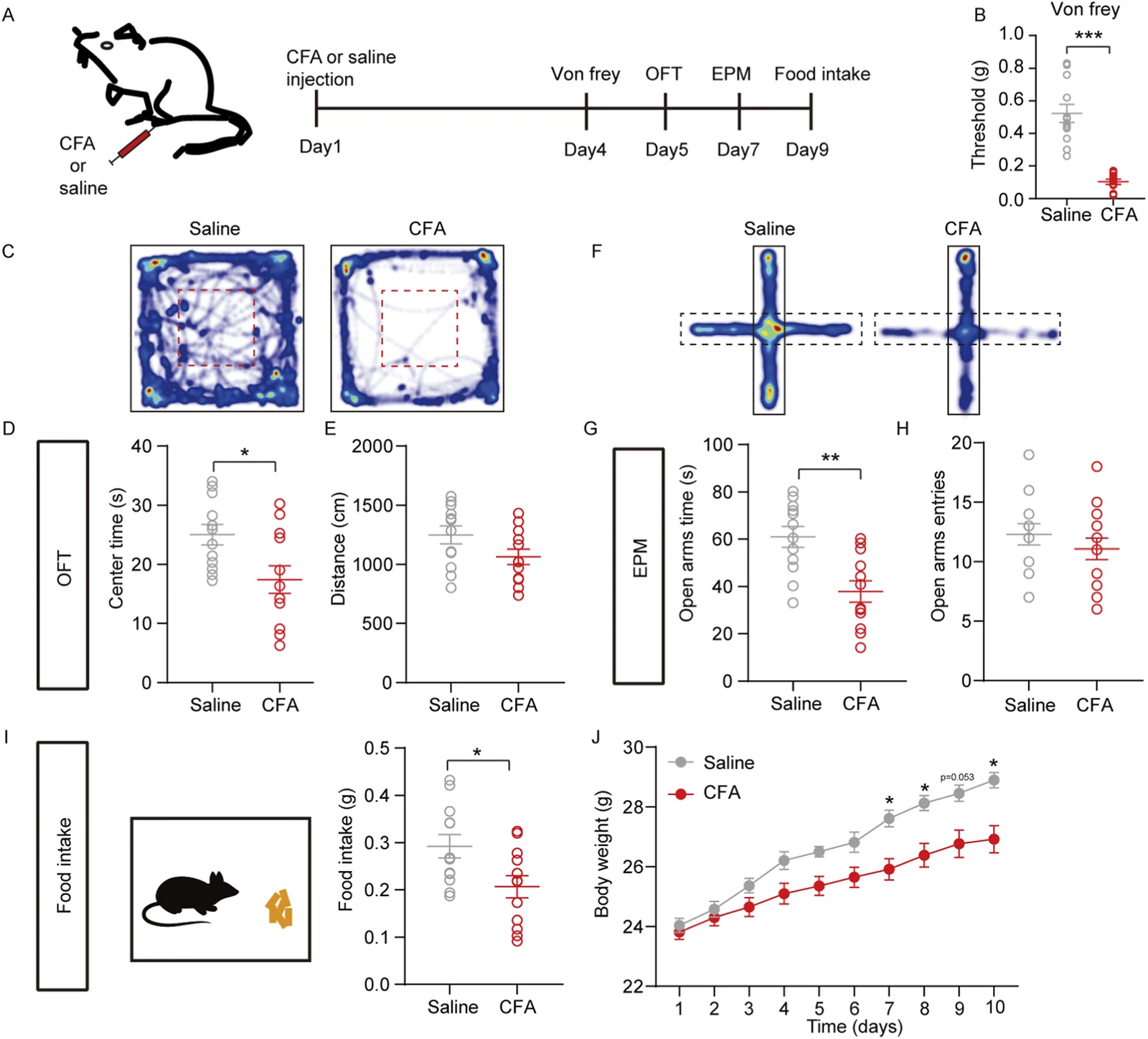
Inflammatory pain caused anxiety-like behavior and decreased appetite in mice. **(A)** CFA injected to the left hind paws of mice to induce inflammation pain (left). Timeline of CFA administration for inflammatory pain and behavioral tests (right). **(B)** The paw withdrawal threshold was assessed using the manual von Frey test in mice with saline control and CFA-induced inflammatory pain (t-test, P < 0.001). **(C)** Heatmaps depicting the locations of control mice and mice experiencing inflammatory pain in the OFT. **(D,E)** represent the central residence time (**(D)**, t-test, P < 0.05) and total distance **(E)**, respectively, of control mice and mice with inflammatory pain in the OFT. **(F)** Heatmaps depicting the locations of control mice and mice experiencing inflammatory pain in the elevated plus maze test (EPM). **(G,H)** Summarized data on the time spent in the open arms (**(G)**, t-test, P < 0.01) and the number of entries into the open arms **(H)** of control mice and mice with inflammatory pain in the EPM. **(I)** Summary of food intake for control mice and mice with inflammatory pain (t-test, P < 0.05). **(J)** Summary plot of the body weight of control mice and mice with inflammatory pain over a 10-day period following saline or CFA injection (one-way ANOVA with repeated measures followed by *post hoc* comparisons between groups. F (9, 198) = 5.276, P < 0.001). 12 mice were in the saline group, and 12 mice were in the CFA group for all the behavior tests.

Inflammatory pain mouse model was established by subcutaneously injecting CFA into the left hind paw (Figure 1A). The CFA mice showed clear signs of allodynia, as evidenced by a significant reduction in mechanical pain thresholds (Figure 1B). To delineate the impacts of inflammatory pain on central neural system function, several behavioral tests were conducted (Figure 1A). In the OFT (Figure 1C), although all the mice traveled a similar distance (Figure 1E), the mice injected with CFA spent less time in the center area of the arena compared to the control mice (Figure 1D). In the EPM (Figure 1F), the mice entered the open arms a similar number of times (Figure 1H). However, the mice that received CFA spent less time in the open arms compared to the control mice (Figure 1G). These results suggest that mice experiencing inflammatory pain exhibit anxiety-like behaviors. In the food intake test (Figure 1I), the CFA mice consumed significantly less food compared to the control mice (Figure 1I). Furthermore, the CFA mice gained less body weight 10 days after CFA injection compared to the saline-injected mice (Figure 1J). These results suggest that inflammatory pain in mice decreased their food appetite.

**FIGURE 2:**
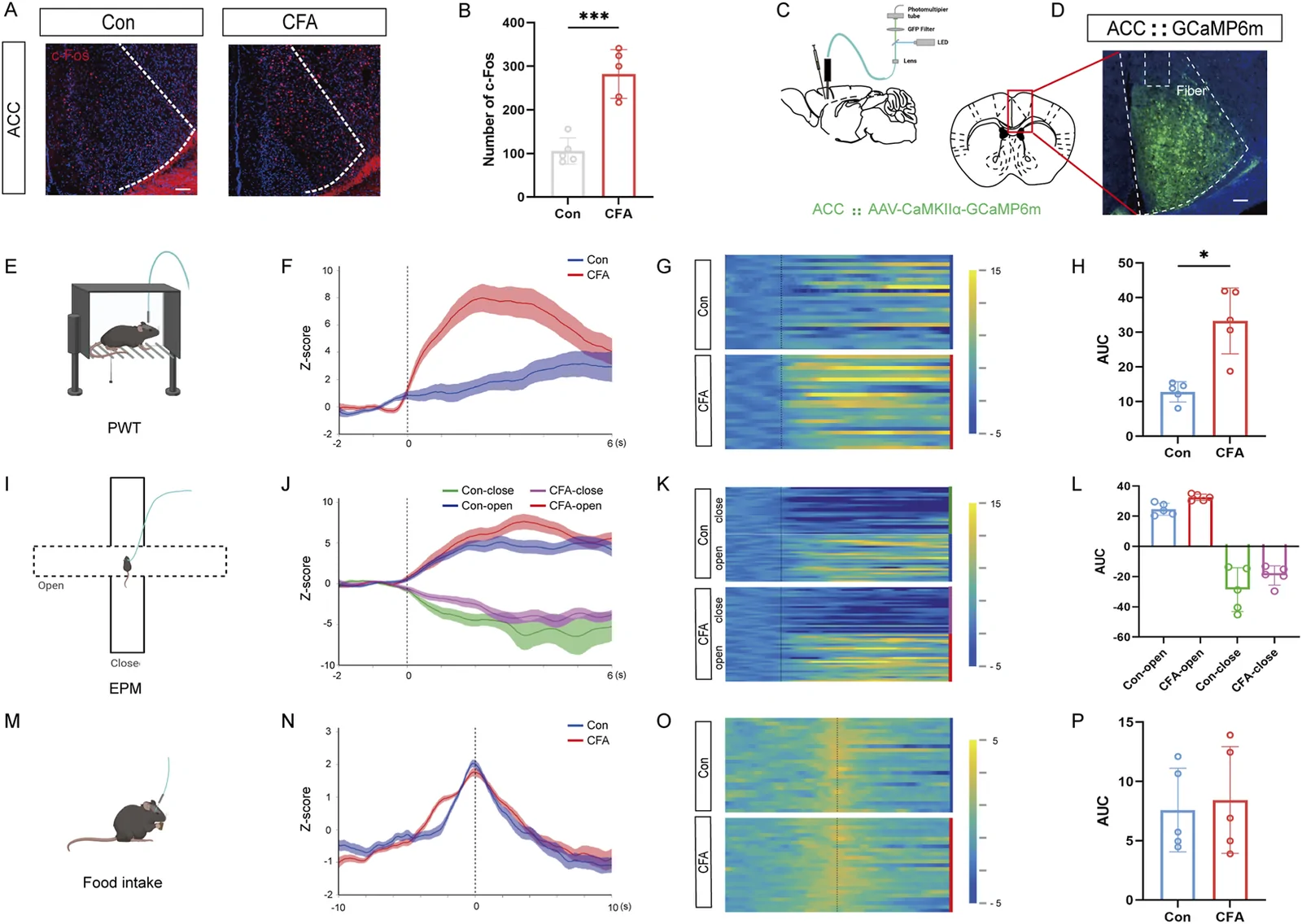
ACC neurons were activated during pain, EPM and food intake. **(A)** Immunofluorescence staining of c-Fos expression in the ACC for both control and CFA groups following mechanical pain stimulation. Scale bars represent 100 μm. **(B)** Statistical analysis of c-Fos expression in the ACC for the control group (5 mice) and CFA group (5 mice, unpaired t-test, P < 0.05). **(C)** Schematic diagram of the *in vivo* calcium signal recording system. **(D)** Representative image of the ACC showing the location of viral injection; scale bar: 100 μm. **(E)** Schematic diagram of mechanical pain stimulations. **(F–H)** Plot of the averaged normalized traces **(F)**, heatmap (**(G)**, 25 trials from 5 mice) and summary **(H)** of Ca2+ transients aligned with the manual von Frey filament stimulations (dotted line, N = 5 mice, t-test, P < 0.05). **(I)** Schematic diagram of EPM. J-L. Plot of averaged normalized traces **(J)**, heatmap (**(K)**, 25 trials from 5 mice) and summary **(L)** of Ca2+ transients when the mice head crossed the line and entered the open or closed arms (dotted line, N = 5 mice, F (3, 16) = 5.484, P < 0.01). **(M)** Schematic diagram of food intake. **(N–P)** Plot of the averaged traces **(N)**, heatmap (**(O)**, 25 trials from 5 mice) and summary **(P)** of Ca2+ transients when the mice began eating (dotted line, N = 5 mice). The color scale on the right indicates ΔF/F (%).

We investigated neural activity following the induction of inflammatory pain in the ACC by observing the expression of the immediate-early gene marker c-Fos. After the mechanical pain stimulation, there are significantly more activated neurons in the ACC of inflammatory pain mice (Figures 2A,B). To investigate the dynamics of ACC neuronal activity in various scenarios, we injected an AAV expressing GCaMP6m into the ACC of mice and recorded calcium signals using photometry system (Figures 2C,D). We observed a rapid increase in calcium signals in the ACC CaMKII neurons of CFA model mice, but not in control mice, in response to mechanical stimuli (Figures 2E–H). The ACC CaMKII neurons exhibited increased activity when the mice entered the open arms (Figures 2I–L). In the food intake test, the ACC CaMKII neurons exhibited calcium transients during feeding onset, with no significant difference relative to control animals (Figures 2M–P).

**FIGURE 3:**
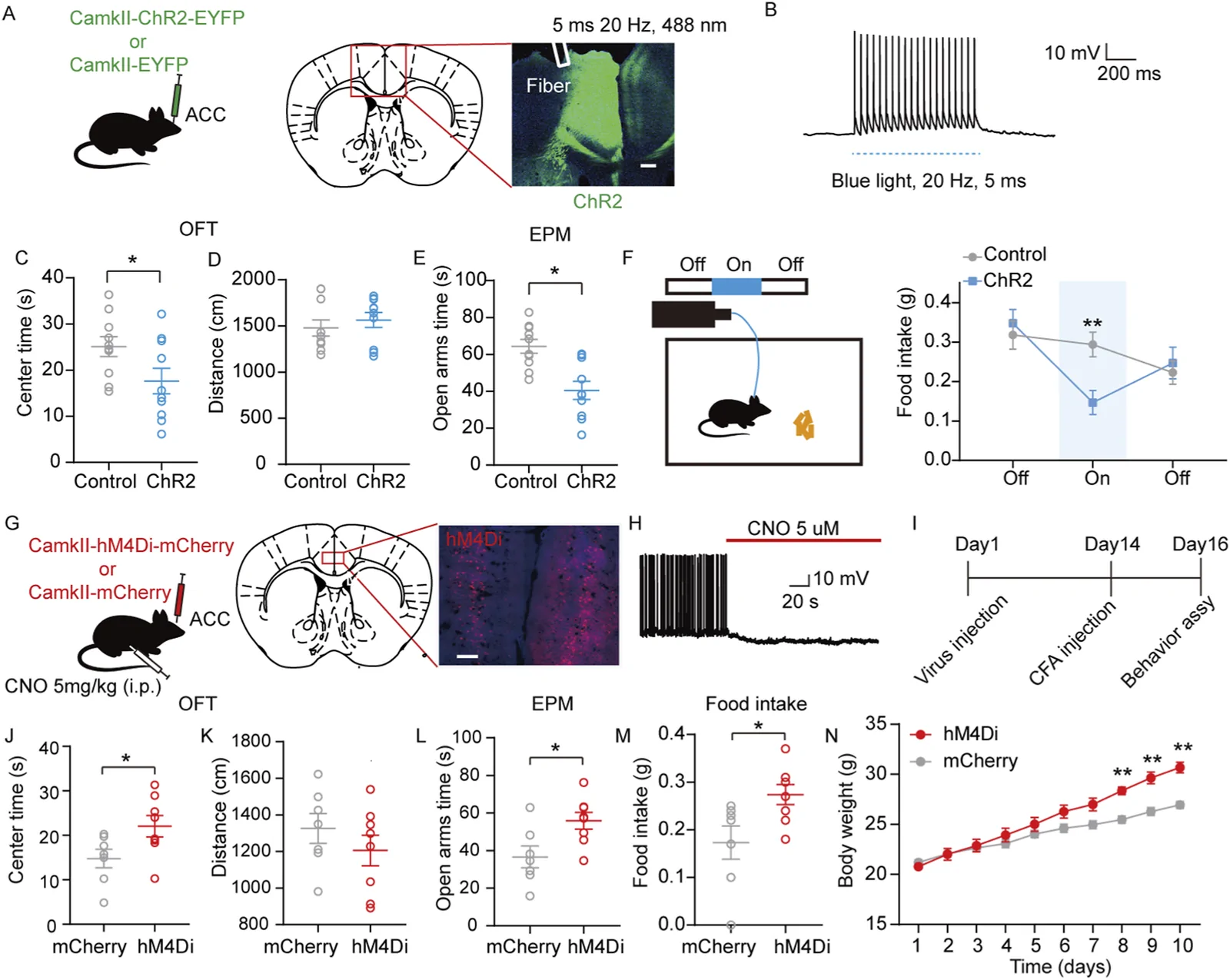
ACC CaMKII neurons involved in anxiety and reduced food intake in mice with inflammatory pain. **(A)** Schematic of ACC injection of AAV-CaMkII-ChR2-EYFP in mice (left). Typical image shows the expression of EYFP in the ACC. Scale bar represents 200 µm. **(B)** Sample trace of action potentials evoked by light (488 nm, 5 ms, 20 Hz, blue line) in an EYFP + neuron in an acute ACC brain slice. **(C)** Reduced the time spent in the center of the OFT through optical activation of ACC CaMKII neurons (P < 0.05, Mann-Whitney U test). **(D)** The total travel distance in the OFT remained unchanged following the optical activation of ACC CaMKII neurons. **(E)** Optical activation of ACC CaMKII neurons decreased the time spent in the open arms of the EPM. *P < 0.05, Mann-Whitney U test. **(F)** Schematic of a food intake test with optical activation of ACC glutamic neurons (left). Food intake decreased after activation (right, n = 10 mice in each group, one-way ANOVA with repeated measures followed by *post hoc* comparisons between groups, F (2, 36) = 5.997, P < 0.01). **(G)** Schematic of ACC injection of AAV-CaMkII-hM4Di-mCherry in mice (left). Typical image shows the expression of mCherry in the ACC. Scale bar represents 100 µm. **(H)** Sample trace of action potentials blocked by CNO (5 μM, red line) in mChrrey + neurons in an acute ACC brain slice. **(I)** Timeline of Virus, CFA injection, and behavioral tests. **(J)** Increased the time spent in the center in the OFT by inhibiting ACC CaMKII neurons in mice with inflammatory pain. P < 0.05, Mann-Whitney U test. **(K)** Inhibition of ACC CaMKII neurons in inflammatory pain mice did not alter the total travel distance in the OFT. **(L)** Increased the open arms time in the EPM by inhibiting ACC CaMKII neurons in mice with inflammatory pain (P < 0.05, Mann-Whitney U test). **(M)** Increased food intake in mice with inflammatory pain after inhibition (P < 0.05, Mann-Whitney U test). **(N)** The body weight increased in the mice with inflammatory pain after inhibition (8 mice were in the hM4Di group, 7 mice were in the mcherry control group. One-way ANOVA with repeated measures followed by *post hoc* comparisons between groups, F (9, 117) = 10.91, P < 0.001).

To investigate whether the activation of ACC is sufficient to induce anxiety-like behavior and appetite in mice, we injected AAV-CaMKIIα-ChR2-EYFP into the ACC in mice (Figure 3A). Blue light evoked action potentials in EYFP-labeled neurons in the ACC of mice injected with ChR2 virus (Figure 3B). The activation of ACC neurons can reduce the time spent in the center area of the arena in the OFT (Figures 3C,D) and in the open arms in the elevated plus-maze test (Figure 3E). The optical activation of ACC neurons can suppress appetite in the food intake test (Figure 3F). These results suggest that activation of the ACC can induce anxiety-like behavior and suppress appetite.

To investigate whether inhibiting the ACC could reverse anxiety-like behavior and appetite suppression induced by inflammatory pain, we injected an AAV-CaMkIIa-hM4Di-mCherry into the ACC of mice (Figure 3G). *Ex vivo* whole-cell recordings of ACC neurons confirmed that bath application of 5 µM CNO can block the spontaneous firing of neurons (Figure 3H). 14 days after injecting viruses, we induced inflammatory pain in mice by injecting CFA (Figure 3I). After the CNO injection, the hM4Di mice spent more time in the central area of the OFT arena compared to the controls (Figure 3J). The total travel distance is similar in the mice of both groups (Figure 3K). In the EPM test, the mice expressing hM4Di spent more time in the open arms compared to the control mice (Figure 3L). In the food intake test, the mice with hM4Di consumed significantly more food compared to the control mice (Figure 3M). The mice with hM4Di gained more body weight 10 days after CFA injection compared to the mice injected with control virus (Figure 3N). These results suggest that chemogenetic inhibition of ACC neurons can suppress anxiety-like behaviors induced by inflammatory pain and increase food appetite.

### The ACC CaMKII→BLA circuit is required for anxiety-like behavior but not suppression of appetite

**FIGURE 4:**
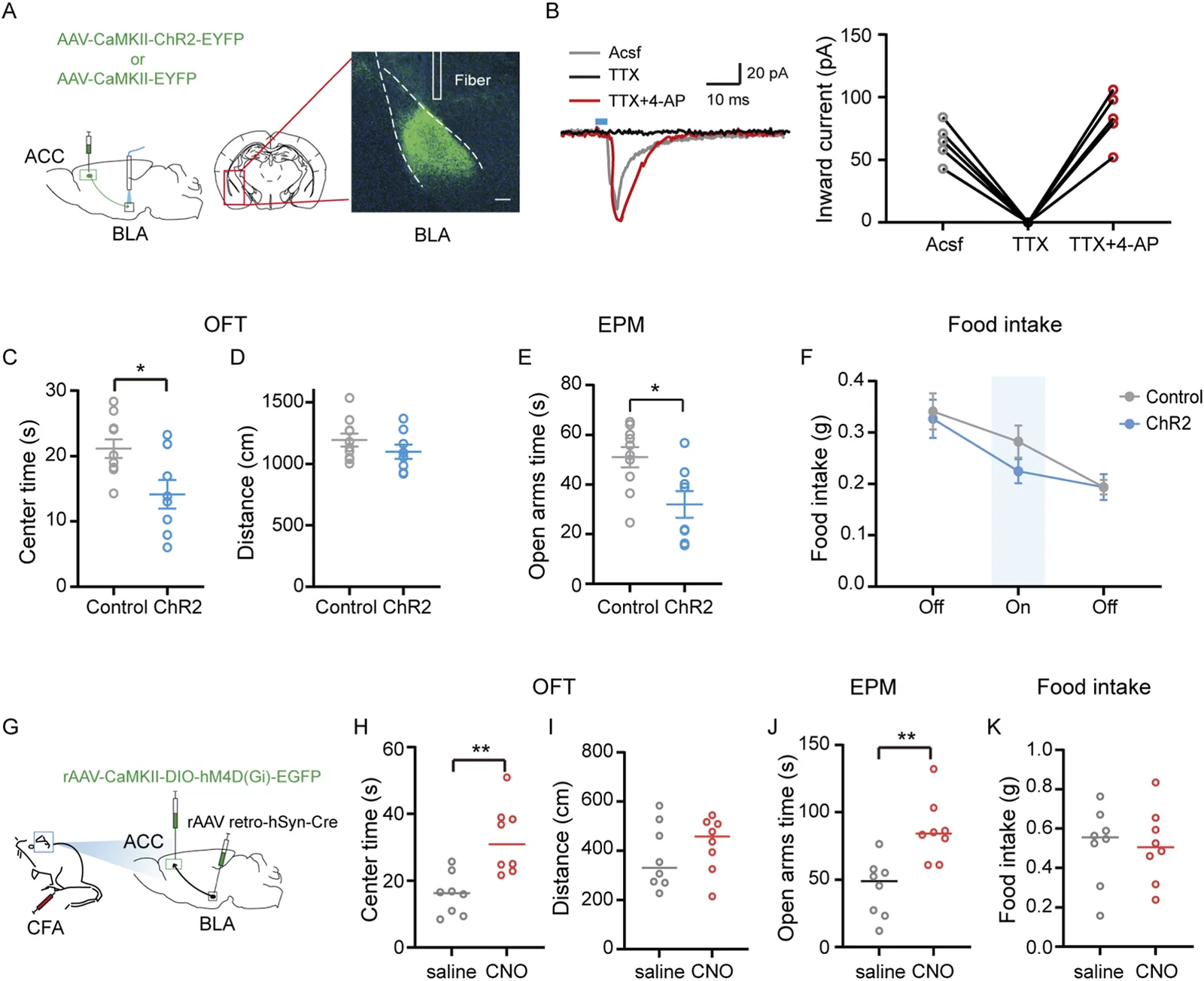
The ACC^CaMKII^→BLA circuit is necessary for modulating anxiety-like behaviors but not food intake. **(A)** Schematic of ACC injection of AAV-CaMkII-ChR2-EYFP into the ACC and implantation of optical cannula into the BLA (left). Representative image of AAV-CaMkII-ChR2-EYFP expression in the BLA (right). Scale bar represents 200 µm. **(B)** Typical traces (left) and summarized data (right, n = 5 cells from 3 mice) of light-evoked postsynaptic current recorded from BLA neurons following optical activation of ACC terminals in ACSF, 1 µM TTX, and 1 μM TTX +200 µM 4-AP. **(C,D)** Decreased the time spent in the center (**(C)**, Mann-Whitney U test, P < 0.05) and left the total travel distance **(D)** unchanged in the OFT through optical activation of the ACC^CaMKII^→BLA pathway. **(E)** Optical activation of the ACC^CaMKII^→BLA pathway decreased the time spent in the open arms in the EPM (P < 0.05, Mann-Whitney U test). **(F)** Unchanged food intake after optical activation of the ACC^CaMKII^→BLA pathway (8 mice were in the ChR2 group, and 10 mice were in the control group. One-way ANOVA with repeated measures, F (2, 32) = 0.5480, P = 0.5834). **(G)** Schematic of AAV retro-hSyn-cre into the BLA and injecting AAV CaMKII-DIO-hM4Di-EFGP into the ACC. **(H,I)** Increased the time spent in the center (**(H)**, Unpaired t-test, P = 0.0017) and unchanged the total travel distance **(I)** in the OFT by chemogenetic inhibiting the ACC^CaMKII^→BLA pathway. **(J)** Increased the open arms time in the EPM by chemogenetic inhibiting the ACC^CaMKII^→BLA pathway (Unpaired t-test, P = 0.0017). **(K)** Unchanged food intake after optically inhibiting the ACC^CaMKII^→BLA pathway (8 mice were in the hM4Di group, and 8 mice were in the control group. Unpaired t-test, P = 0.18).

In order to examine the roles of projection-specific circuits in anxiety-like behavior and appetite suppression, we first targeted the ACC^CaMKII^→BLA circuit. ChR2 was virally expressed in the ACC under the control of the CaMKIIα promoter (Figure 4A). Voltage-clamp recording of brain slices revealed that stimulation of ChR2-positive fibers with blue light in the BLA can evoke EPSC in the BLA neurons (Figure 4B). To investigate the impact of activating the ACC^CaMKII^→BLA circuit on anxiety-like behaviors and appetite in mice, we implanted an optic cannula into the BLA (Figure 4A). We found that activation of the ACCCaMKII→BLA circuit decreased the time spent in the center area in the OFT (Figure 4C), without affecting the total travel distance (Figure 4D), and decreased the time spent in the open arms in the EPM (Figure 4E). The blue light stimulation had minimal effects on food intake tests (Figure 4F). These results indicate that optical activation of the ACC^CaMKII^→BLA circuit promotes anxiety-like behaviors with minimal impact on appetite.

To investigate the impact of inhibiting the ACC^CaMKII^→BLA circuit on anxiety-like behaviors and appetite in CFA mice, we injected AAV retro-hSyn-cre into the BLA and AAV CaMKII-DIO-hM4Di-EFGP into the ACC (Figure 4G). Inhibiting the ACC^CaMKII^→BLA circuit in CFA injected mice with CNO increased the time spent in the center area in the OFT (Figures 4H,I) and the time spent in the open arms in the EPM (Figure 4J). The CNO induced minimal effects on food intake tests (Figure 4K). The combination of optic activation and chemogenetic inhibition results suggest that the ACC^CaMKII^→BLA circuit plays a vital role in anxiety-like behaviors but not in appetite.

### The ACC^CaMKII^→NAc circuit is necessary for suppressing appetite but not for inducing anxiety-like behavior

**FIGURE 5:**
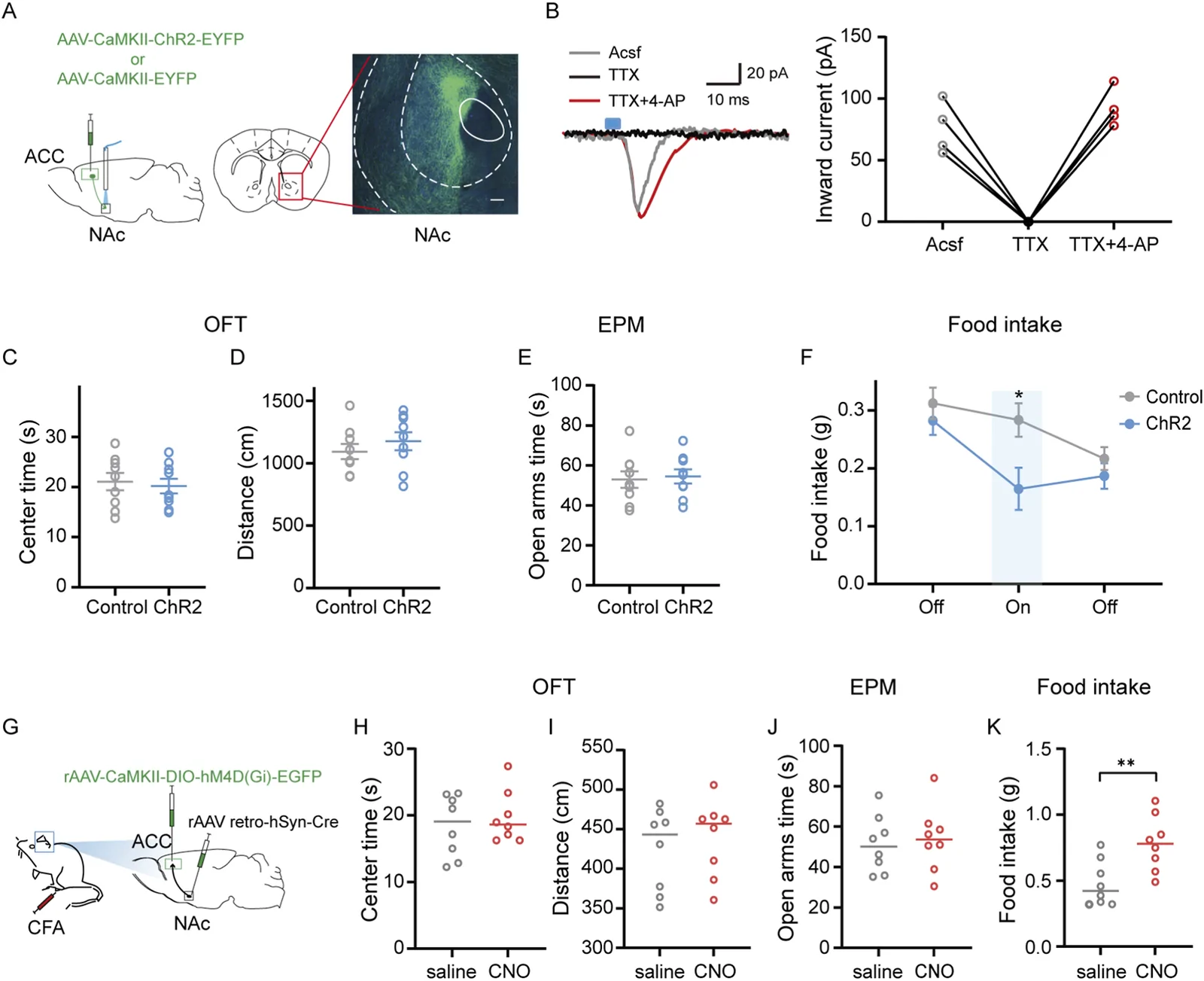
The ACC^CaMKII^→NAc circuit is necessary for regulating food intake but not anxiety-like behaviors. **(A)** Schematic of the ACC injection of AAV-CaMKII-ChR2-EYFP into the ACC and the implantation of an optical cannula into the NAc (left). Representative image of AAV-CaMKII-ChR2-EYFP expression in the NAc (right). Scale bar represents 200 µm. **(B)** Typical traces (left) and summarized data (right, n = 4 cells from 3 mice) of light-evoked postsynaptic current recorded from NAc neurons following optical activation of ACC terminals in ACSF, 1 µM TTX, and 1 μM TTX +200 µM 4-AP. C-D. Unchanged the time at the center **(C)** and the total travel distance **(D)** in the OFT through optical activation of the ACC^CaMKII^→NAc pathway. **(E)** Uncahnged the open arms time in the EPM through optical activation of the ACC^CaMKII^→NAc pathway. P = 0.77, Mann-Whitney U test. **(F)** Food intake decreased after optical activation of the ACC^CaMKII^→NAc pathway (n = 10 mice in each group. One-way ANOVA with repeated measures followed by *post hoc* comparisons between groups, F (2, 32) = 2.008, P < 0.05). **(G)** Schematic of ACC injection of AAV retro-hSyn-cre into the NAc and injecting AAV CaMKII-DIO-hM4Di-EFGP into the ACC. **(H,I)** Maintained the time in the center **(H)** and the total travel distance **(I)** in the OFT by chemogenetic inhibiting the ACC^CaMKII^→NAc pathway. **(J)** Unchanged the open arms time in the EPM by optically inhibiting the ACC^CaMKII^→NAc pathway. **(K)** Increased food intake after chemogenetic inhibiting the ACC^CaMKII^→NAc pathway (8 mice were in the hM4Di group, and 8 mice were in the control group. Unpaired t-test, P = 0.0069).

In order to investigate the roles of the ACC^CaMKII^→NAc circuit in anxiety-like behavior and appetite suppression. We injected the ChR2-expressing and EYFP control viruses into the ACC and implanted an optic cannula into the NAc (Figure 5A). Voltage clamp recordings on NAc brain slices revealed monosynaptic inputs from the ACC to NAc neurons (Figure 5B). Optical activation of the ACC^CaMKII^→NAc circuit had minimal effect in the OFT and the EPM (Figures 5C–E). However, the activation of blue light could decrease food intake in mice expressing ChR2 (Figure 5F). However, the chemogenetic inhibition of the ACC^CaMKII^→NAc circuit with hM4Di expression in CFA injected mice had little effect on OFT, EPM (Figures 5G–J), but increased the food intake (Figure 5K). Together, these results suggest that the ACC^CaMKII^→NAc circuit plays an important role in appetite suppression but not in anxiety-like behaviors.

### Inflammatory pain shifts the excitatory-inhibitory balance of ACC^CaMKII^→BLA and ACC^CaMKII^→NAc neuronal activity towards excitation distinctly

**FIGURE 6:**
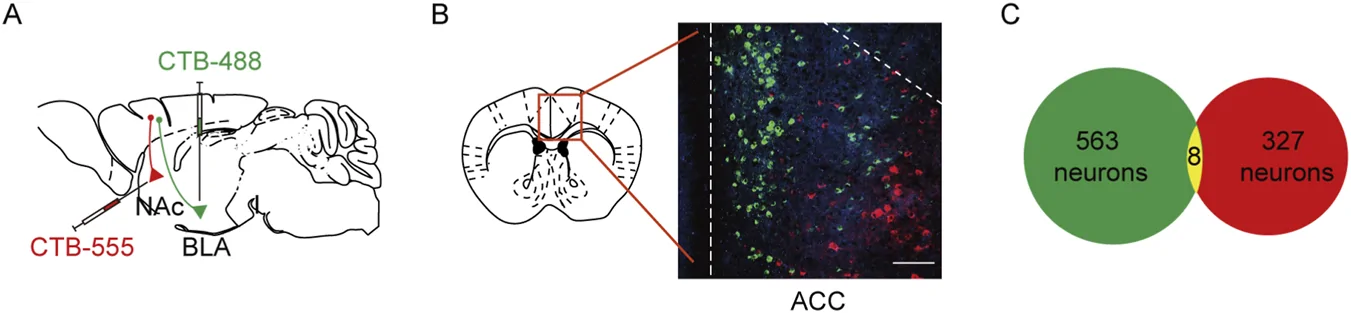
The ACC neurons projecting to the NAc and BLA are distinct. **(A)** Schematic of the injection of cholera toxin-B Alexa Fluor-555 (CTB-555) and cholera toxin-B Alexa Fluor-488 (CTB-488) into the NAc and BLA. **(B)** Retrograde labeling of the ACC→NAc and ACC→BLA neurons. Scale bar represent 100 µm. **(C)** Cell counts of ACC cells positive for CTB-555 (red) or CTB-488 (green). Double-labeled cells yellow. 16 slices from 4 mice.

To isolated the ACC→BLA and ACC→NAc neurons, we injected retrograde neuroanatomical tracer Alexa Fluor 488-conjugated cholera toxin subunit B (CTB) into the BLA and Alexa Fluor 555–CTB into the NAc (Figure 6A). We found that the neurons projecting to the BLA and NAc in the ACC were largely distinct (Figures 6B,C). This indicates that two distinct ACC neuronal populations can directly project to the BLA and NAc.

**FIGURE 7:**
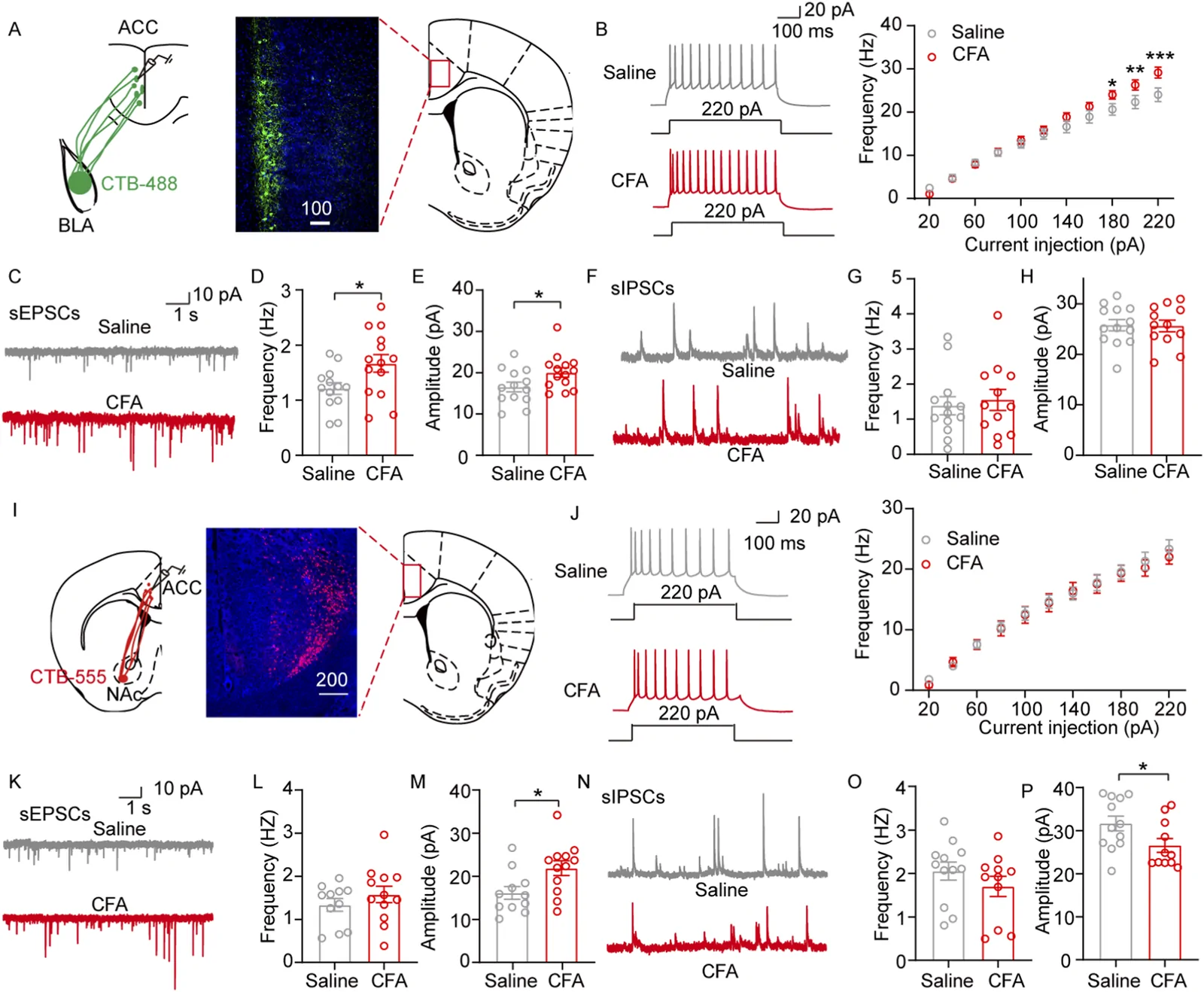
Inflammatory pain modulates the excitability and synaptic transmission of ACC^CaMKII^→BLA neurons and ACC^CaMKII^→NAc neurons differently. **(A)** schematic for CTB-488 injection in the BLA and whole-cell recording in acute ACC brain slices (left). Representative image of CTB-488 labeled cells in the ACC of mice (right). The scale bar represents 100 µm. **(B)** Representative action potential traces (left) and summarized data (right) recorded in ACC^CaMKII^→BLA neurons from the specified group (n = 13 cells from 7 mice in saline group, n = 14 cells from 5 mice in CFA group, one-way ANOVA with repeated measures followed by *post hoc* comparisons between groups, F (10, 250) = 6.866, P < 0.0001). **(C–E)** Representative traces of sEPSC **(C)** and summarized frequency (**(D)**, P < 0.05, Mann-Whitney U test) and amplitude (**(E)**, P < 0.05, Mann-Whitney U test) recorded in ACC^CaMKII^→BLA neurons from the indicated group (n = 13 cells 3 mice in saline group, n = 14 cells from 4 mice in CFA group). **(F–H)** Representative traces of sIPSC **(F)** and summarized frequency (G, P > 0.05, Mann-Whitney U test) and amplitude (H, P > 0.05, Mann-Whitney U test) recorded in ACC^CaMKII^→BLA neurons from the indicated group are shown (n = 13 cells from 3 mice in saline group, n = 12 cells from 3 mice in CFA group). **(I)** Schematic for CTB-555 injection in the NAc and whole-cell recording in acute ACC brain slices (left). Representative image showing CTB-555 labeled cells in the ACC of mice (right). The scale bar represents 200 µm. **(J)** Representative action potential traces (left) and summarized data (right) recorded in ACC^CaMKII^→NAc neurons from the specified group (n = 9 cells from 3 mice in each group, one-way ANOVA with repeated measures, F (10, 160) = 0.5483, P = 0.8535). **(K–M)** Representative traces of sEPSC **(K)** and summarized frequency (**(L)**, P > 0.05, Mann-Whitney U test) and amplitude (**(M)**, P < 0.05, Mann-Whitney U test) recorded in ACC^CaMKII^→NAc neurons from the indicated group (n = 11 cells from 3 mice in saline group, n = 12 cells from 3 mice in CFA group). **(N–P)** Representative traces of sIPSC **(N)** and summarized frequency (**(O)**, P > 0.05, Mann-Whitney U test) and amplitude (**(P)**, P < 0.05, Mann-Whitney U test) recorded in ACC^CaMKII^→NAc neurons from the indicated group (n = 12 cells from 3 mice in saline group, n = 11 cells from 3 mice in CFA group). *P < 0.05, **P < 0.01, ***P < 0.001.

Given established subregional differences in pain-related receptor expression between ACC cg1 and cg2 (D2R and M2R divergence) (Ortega-Legaspi et al., 2011; Ortega-Legaspi et al., 2010), we quantified ACC neurons projecting to the BLA and NAc across 16 brain slices from four mice. No significant inter-subregion differences were observed: ACC-BLA counts were 318 (cg1) vs. 207 (cg2, P = 0.37), and ACC-NAc counts were 93 (cg1) vs. 121 (cg2, P = 0.48; unpaired t-tests). We next analyzed laminar distribution of these projection populations (representative micrographs in Figures 6, 7). ACC→BLA neurons showed balanced superficial (230) and deep-layer (183) localization (P = 0.6), while ACC→NAc neurons displayed a qualitative bias toward deep layers (76 superficial, 138 deep) that did not achieve statistical significance (P = 0.22). Together, anatomical quantification revealed no statistically robust subregional or laminar segregation of ACC-BLA and ACC-NAc projection neuron populations.

To investigate the mechanisms underlying changes in region-specific projecting ACC neuronal activity in chronic inflammatory pain. We conducted whole-cell electrophysiological recording of pyramidal neurons in the ACC brain slices. In the brain slice with CTB-488 injected into the BLA (Figure 7A), the firing frequencies of ACC^CaMKII^→BLA neurons in response to step-increasing currents were higher in CFA mice compared to control mice (Figure 7B). We also discovered that the frequency and amplitude of sEPSCs of ACC^CaMKII^→BLA neurons were significantly increased in CFA mice compared to those in sham mice (Figures 7C–E). In contrast, the frequency and amplitude of sIPSCs were not altered by inflammatory pain (Figures 7F–H). The results indicate that the intrinsic excitability and excitatory synaptic inputs in ACC^CaMKII^→BLA neurons were increased in mice experiencing inflammatory pain.

Regarding to ACC^CaMKII^→NAc neurons, their firing frequencies were not affected by inflammatory pain (Figures 7I,J). Subsequently, we analyzed the excitatory and inhibitory synaptic inputs to ACC^CaMKII^→NAc neurons. The amplitudes of sEPSCs and sIPSCs changed in opposite directions with inflammatory pain (Figures 7K–P). The amplitude of sEPSCs increased, while there was a decrease in sIPSCs amplitude (Figures 7M,P). Together, the opposing changes in excitatory and inhibitory transmission suggested the net increase in excitatory drive for ACC^CaMKII^→NAc neurons with inflammatory pain. Complementing our findings, a previous study from the Sohal group demonstrated that transplantation of GABAergic neurons within the ACC produces robust analgesic effects (Juarez-Salinas et al., 2019), further supporting the model that chronic inflammatory pain shifts the excitatory–inhibitory synaptic balance toward heightened excitation in the ACC.

## Discussion

Understanding the circuit mechanisms underlying the complex negative symptoms of inflammatory pain is a crucial step in developing new effective treatments. Here, we find that the ACC projecting to the BLA and NAc circuits contribute to anxiety-like behavior and appetite suppression, respectively, in inflammatory pain. We also found that the neurons projecting from the ACC to these two brain regions are largely distinct. Furthermore, the two populations of the ACC neurons are differentially altered under inflammatory pain.

Feeding behavior is essential for survival and is governed by multiple brain regions13, including the homeostatic and hedonic feeding pathways (Marinescu and Labouesse, 2024). The homeostatic pathway is primarily located in the hypothalamus. The lateral hypothalamus (LH), has been shown to mediate appetite suppression in inflammatory pain (Tang et al., 2022). The hedonic feeding circuit, on the other hand, is closely linked to the reward system, particularly the dopamine system (Baik, 2021). The NAc is predominantly composed of inhibitory neurons, most of which express dopamine receptors D1 and D2 (Bertran-Gonzalez et al., 2008). During food-seeking behavior, only NAc neurons expressing the D1 receptor are activated (Matikainen-Ankney et al., 2023). Activation of these D1-positive neurons has been shown to inhibit food intake (Walle et al., 2024). Furthermore, NAc D1 neurons project to GABAergic neurons in the LH (Diepenbroek et al., 2024; Yonemochi et al., 2019). Here, we found that the ACC→NAc pathway can modulate appetite in inflammatory pain. Together with the previously reported ACC→LH^CaMKII^ pathway (Tang et al., 2022), our results suggest that the ACC may suppress food intake through two distinct mechanisms: directly by exciting excitatory neurons in the LH, and indirectly by disinhibiting LH GABAergic neurons via the NAc.

The BLA is the primary output region of the amygdala and is considered to be a crucial brain area involved in anxiety and fear memory (Luo et al., 2020). BLA received input from many different brain areas. Rapid inhibition of the BLA neurons leads to an immediate decrease in anxiety-like behavior in male mice (Munshi et al., 2023). Chemical genetic inhibition of the projection from the Locus Coeruleus (LC) to the BLA can alleviate anxiety-like behaviors induced by pain (Llorca-Torralba et al., 2019). Here, we found that the ACC→BLA circuit modulates anxiety-like behaviors induced by pain. Elevated activity of the prelimbic cortex→BLA circuit has been reported in studies of stress models, including chronic restraint stress, acute social defeat stress, chronic ethanol exposure and withdrawal, and chronic pain-induced anxiety-like behaviors (Gao et al., 2023; Grossman et al., 2022; Liu et al., 2020). The inputs to the BLA are known to regulate anxiety-like behavior with varying effects. Both the ACC and the LC project to the BLA and are involved in pain-induced anxiety. In pain-free animals, activation of the LC→BLA pathway elicits anxiety-like behavior, whereas its inhibition does not affect baseline anxiety (Llorca-Torralba et al., 2019; McCall et al., 2017). In contrast, we found that the ACC→BLA pathway can bidirectionally regulate anxiety. Furthermore, functional heterogeneity exists in BLA outputs based on anatomical specificity. For instance, activation of the posterior BLA→CA1 pathway has an anxiolytic effect, while activation of the anterior BLA→CA1 pathway is anxiogenic (Liu et al., 2020). Similarly, posterior and anterior BLA projections to the bed nucleus of the stria terminalis (BNST) oppositely regulate anxiety (Han et al., 2024). Given that numerous brain areas converging on the BLA regulate anxiety in chronic pain, future research should dissect the specific target neuron populations within these discrete circuits that mediate inflammatory pain-induced anxiety.

Activation of the ACC has also been associated with emotional or psychological co-morbidities of chronic pain (Rolls, 2023; Zhuo, 2006). Recently, ACC neurons have been found to serve as a critical node for encoding safety/danger assessment and mediating behavioral flexibility through differential circuits (Wu et al., 2023). The precise organization and functions of ACC cell types remain largely unknown. Here, we identified two circuits: ACC^CaMKII^→BLA and ACC^CaMKII^→NAc, which can separately modulate pain-induced anxiety-like behavior and appetite suppression. More interestingly, the ACC neurons projecting to the BLA and NAc are largely distinct. These results suggest that specific subpopulations of ACC neurons mediate particular pain behaviors, which helps clarify how discrete circuits underlie specific comorbidities associated with chronic pain.

Hyperactivation of the ACC brain regions has been detected in functional MRI studies of patients with neuropathic pain and chronic visceral pain (Bliss et al., 2016). The mechanisms of hyperactivation of ACC neurons include elevated intrinsic excitability, increased excitatory synaptic input (Zhao et al., 2018), decreased dopaminergic inhibition (Lançon et al., 2021), the expression of LTP (Koga et al., 2015) and suppression of LTD induction (Bliss et al., 2016; Wang et al., 2020). Whether subpopulations of ACC neurons undergo specific changes after chronic pain is still unclear. Here, we observed the increased intrinsic excitability and excitatory synaptic inputs in ACC^CaMKII^→BLA neurons, while there were opposing changes in excitatory and inhibitory transmission in ACC^CaMKII^→NAc neurons in chronic inflammatory pain mice. The various changes in ACC^CaMKII^→BLA and ACC^CaMKII^→NAc neurons following chronic inflammatory pain help us to selectively modify the excitability of certain neuron types, paving the way for the development of targeted therapies to alleviate specific co-morbidities associated with chronic inflammatory pain.

Several limitations constrain the interpretability of our anatomical and functional circuit findings. A notable mismatch exists between pan-ACC population calcium activity and projection-specific behavioral effects on feeding and anxiety; restricted experimental resources prevented us from conducting projection-specific calcium recordings to disentangle BLA-versus NAc-projecting ACC neuronal dynamics. Furthermore, we omitted pain behavioral measurements during circuit manipulations. Prior work confirms ACC sends functionally distinct projections to multiple downstream targets including PAG and STN specifically transmits affective pain signals (Wang et al., 2024; Choi et al., 2025), while ACC→BLA and ACC→NAc pathways exerting minimal effects on mechanical nociception (Smith et al., 2021; Corder et al., 2019). While we focused on feeding and anxiety-like behaviors, the lack of pain readouts limits analysis of pain-emotion comorbidity mechanisms mediated by these two circuits. Lastly, our moderate anatomical sample size may lack power to detect subtle subregional or laminar projection biases. Follow-up work will employ subregion-restricted viral tools, projection-specific calcium imaging, full pain-behavior batteries, and larger animal cohorts to resolve these caveats.

## Data Availability

The raw data supporting the conclusions of this article will be made available by the authors, without undue reservation.
