# Supplementary material for: Distinct CaMKII neurons in anterior cingulate cortex regulate anxiety-like behavior and appetite suppression in a model of inflammatory pain
Source: Front Pharmacol. 2026 Sep 3;17:1897103. doi: 10.3389/fphar.2026.1897103 (PMC13603023; doi:10.3389/fphar.2026.1897103)
Supplement: Supplementary file 1 [file DataSheet1.pdf]

FIGURE 29

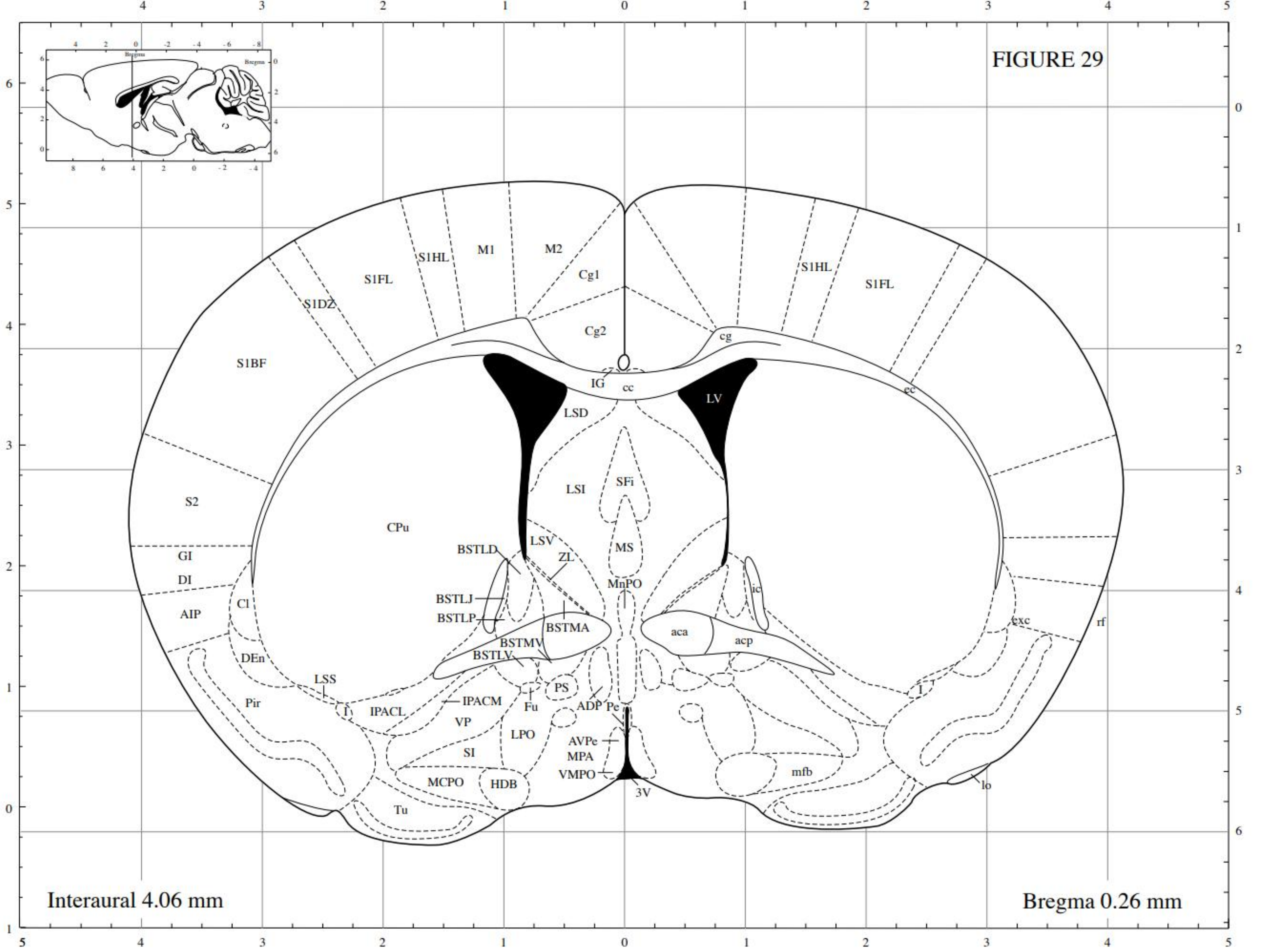

Interaural 4.06 mm

Bregma 0.26 mm

1eg. acc

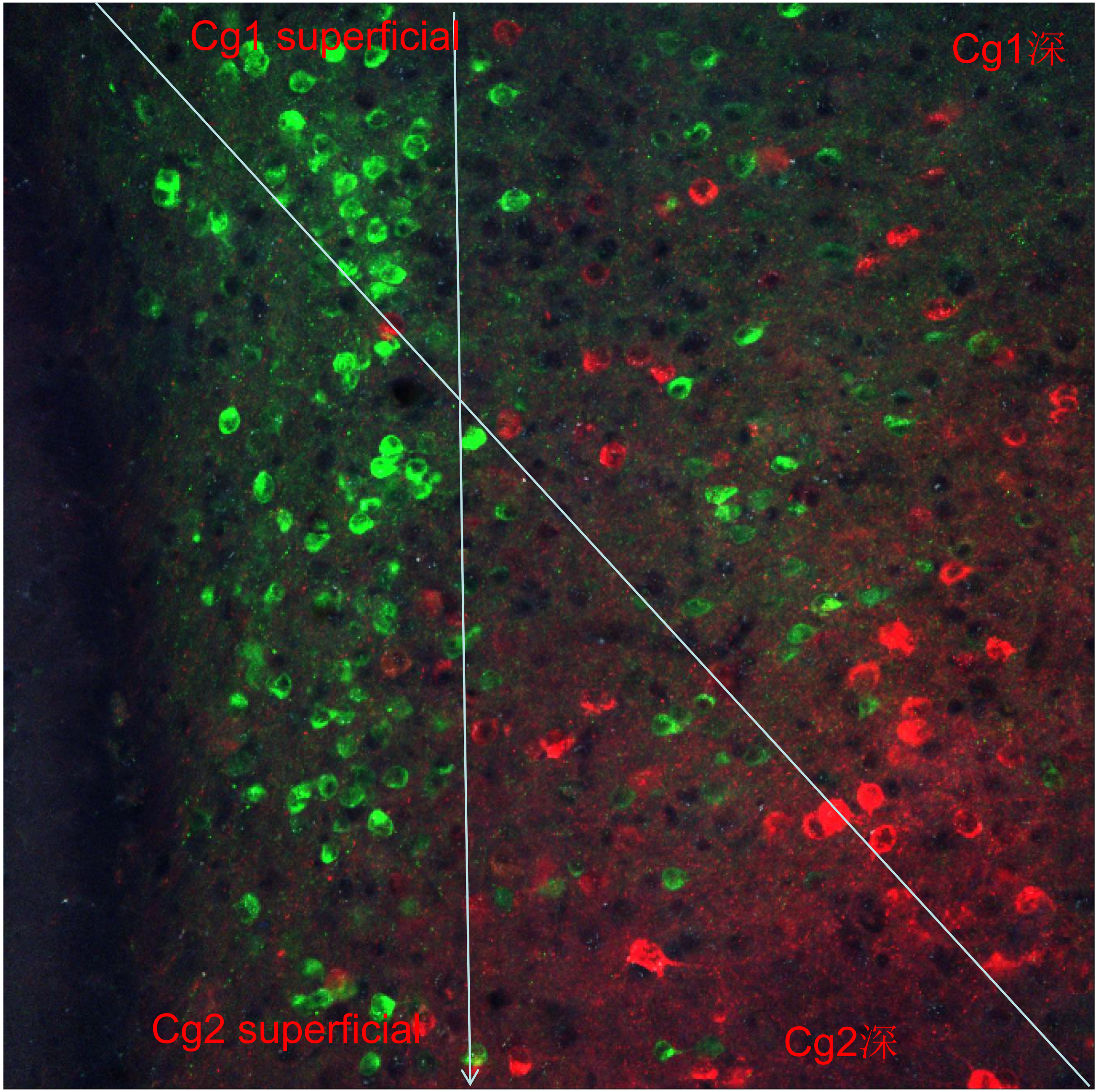

green：
Cg1 superficial：24
Cg1 deep：34
Cg2 superficial：62
Cg2 deep：18

red：
Cg1 superficial：1
Cg1 deep：37
Cg2 superficial：7
Cg2 deep：16

2

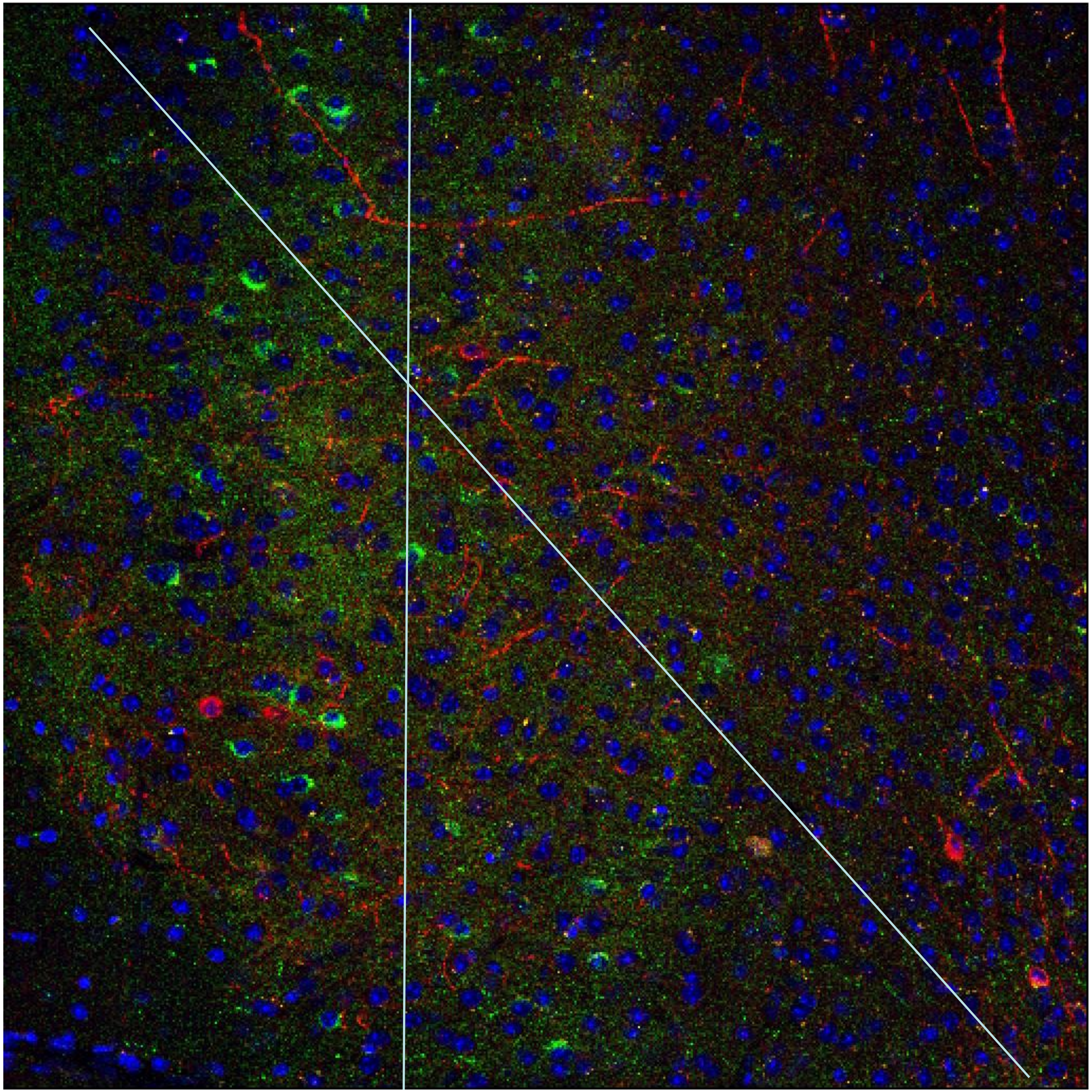

green：
Cg1 superficial：5
Cg1 deep：5
Cg2 superficial：11
Cg2 deep：9

red：
Cg1 superficial：0
Cg1 deep：4
Cg2 superficial：6
Cg2 deep：3

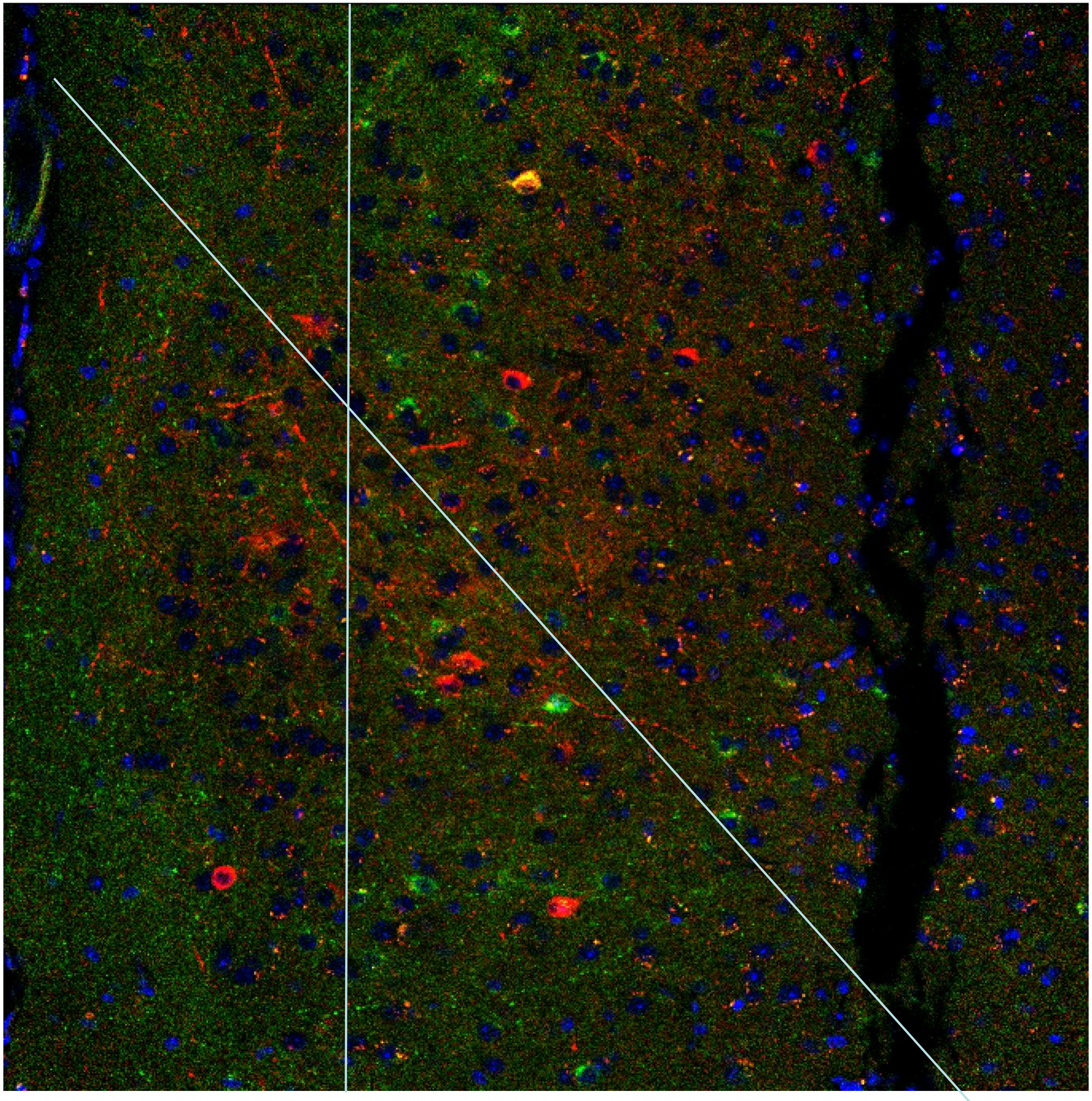

green：
Cg1 superficial：0
Cg1 deep：11
Cg2 superficial：5
Cg2 deep：6

red：
Cg1 superficial：1
Cg1 deep：5
Cg2 superficial：3
Cg2 deep：3

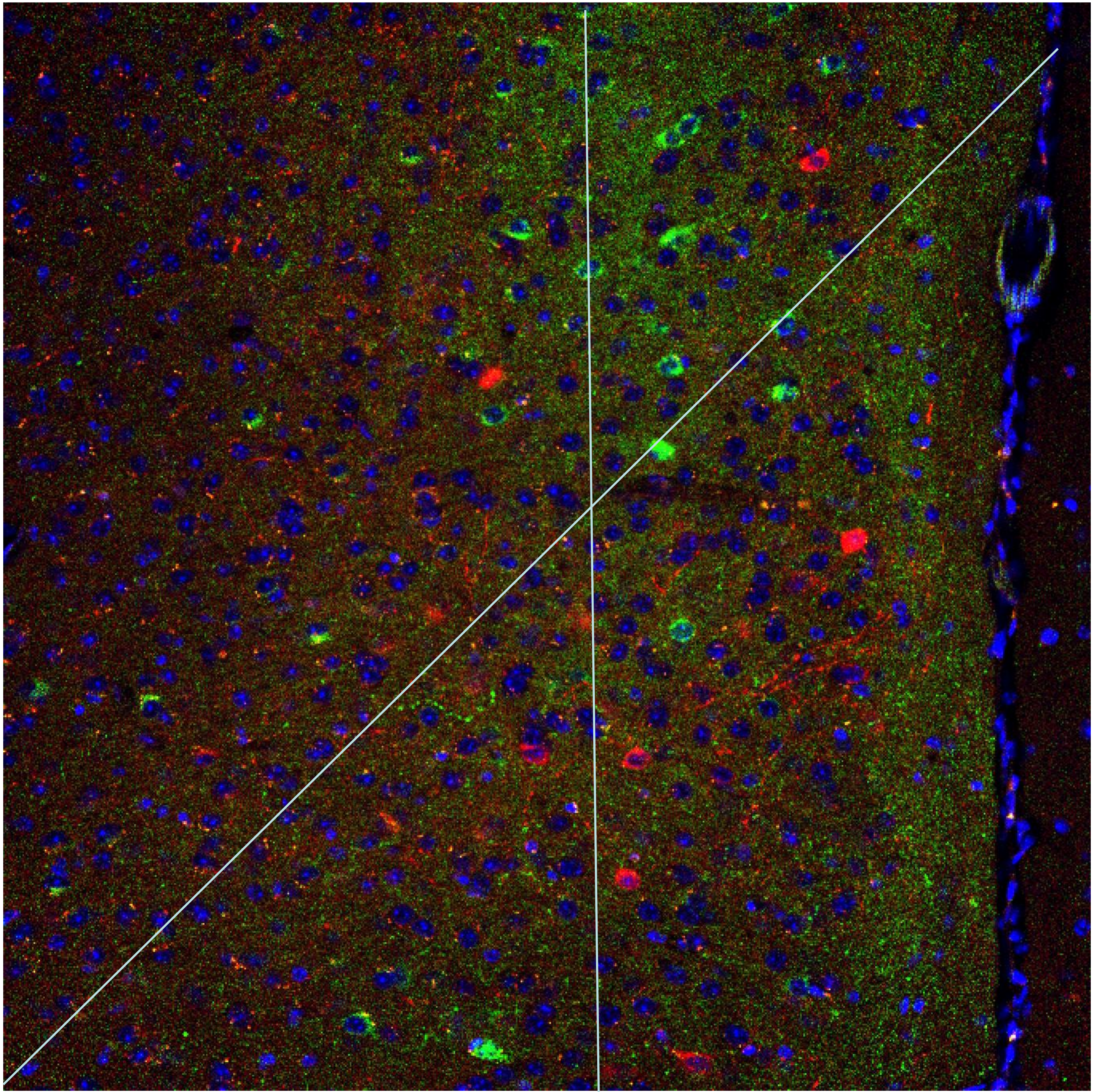

green：
Cg1 superficial：7
Cg1 deep：11
Cg2 superficial：4
Cg2 deep：3

red：
Cg1 superficial：1
Cg1 deep：2
Cg2 superficial：4
Cg2 deep：3

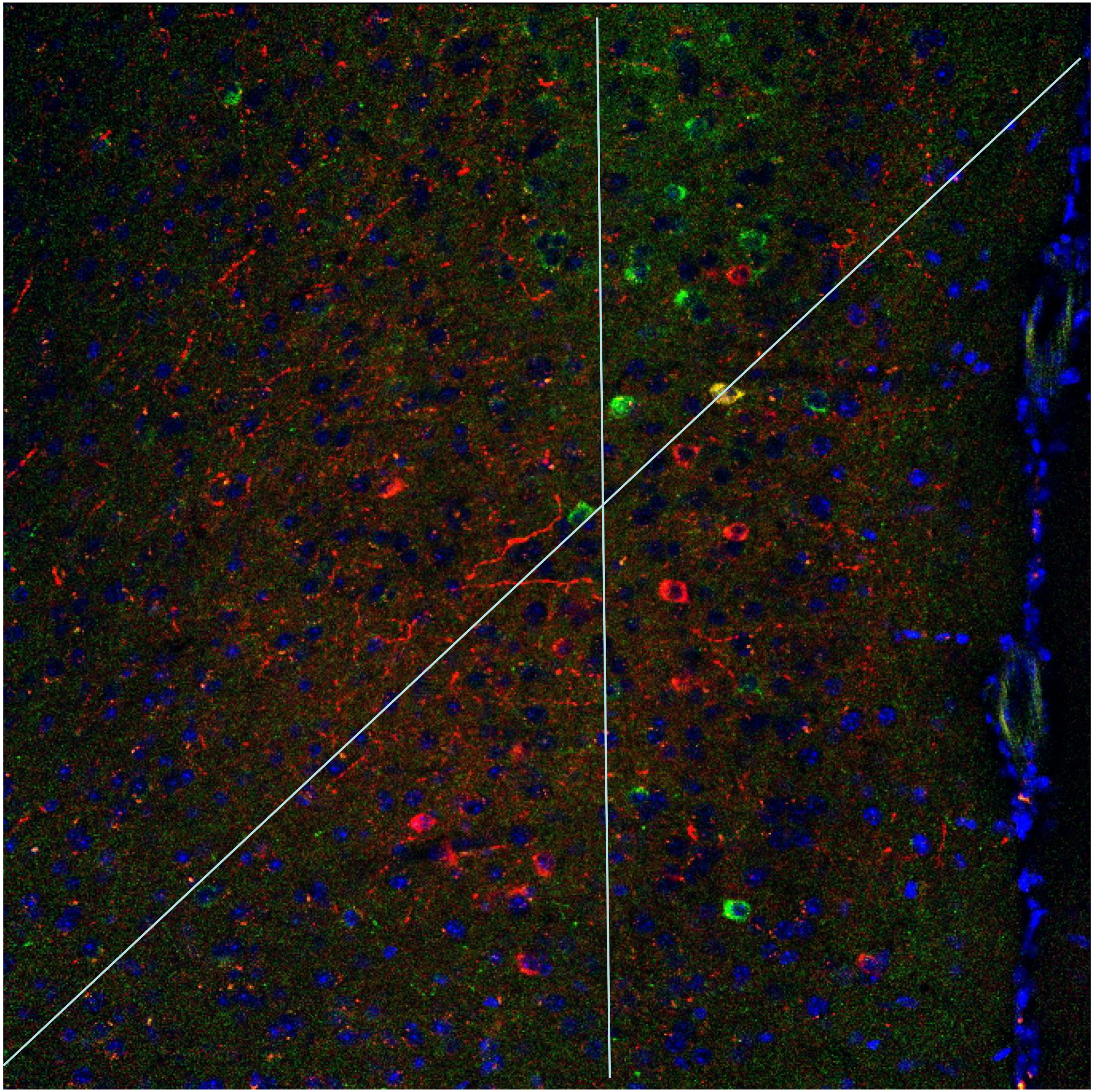

green：
Cg1 superficial：8
Cg1 deep：3
Cg2 superficial：5
Cg2 deep：0

red：
Cg1 superficial：4
Cg1 deep：4
Cg2 superficial：6
Cg2 deep：8

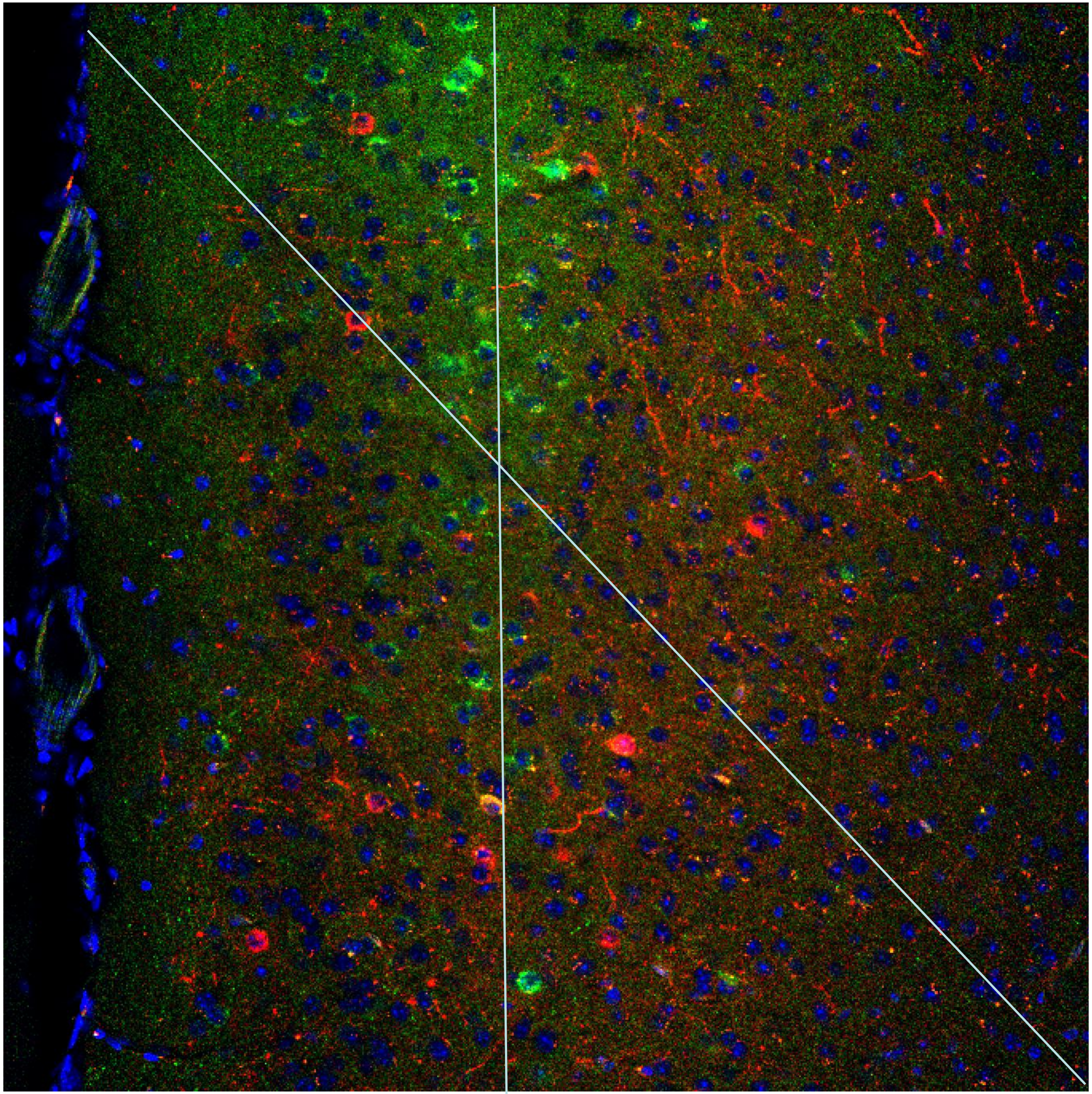

green：
Cg1 superficial：12
Cg1 deep：11
Cg2 superficial：5
Cg2 deep：3

red：
Cg1 superficial：2
Cg1 deep：2
Cg2 superficial：6
Cg2 deep：5

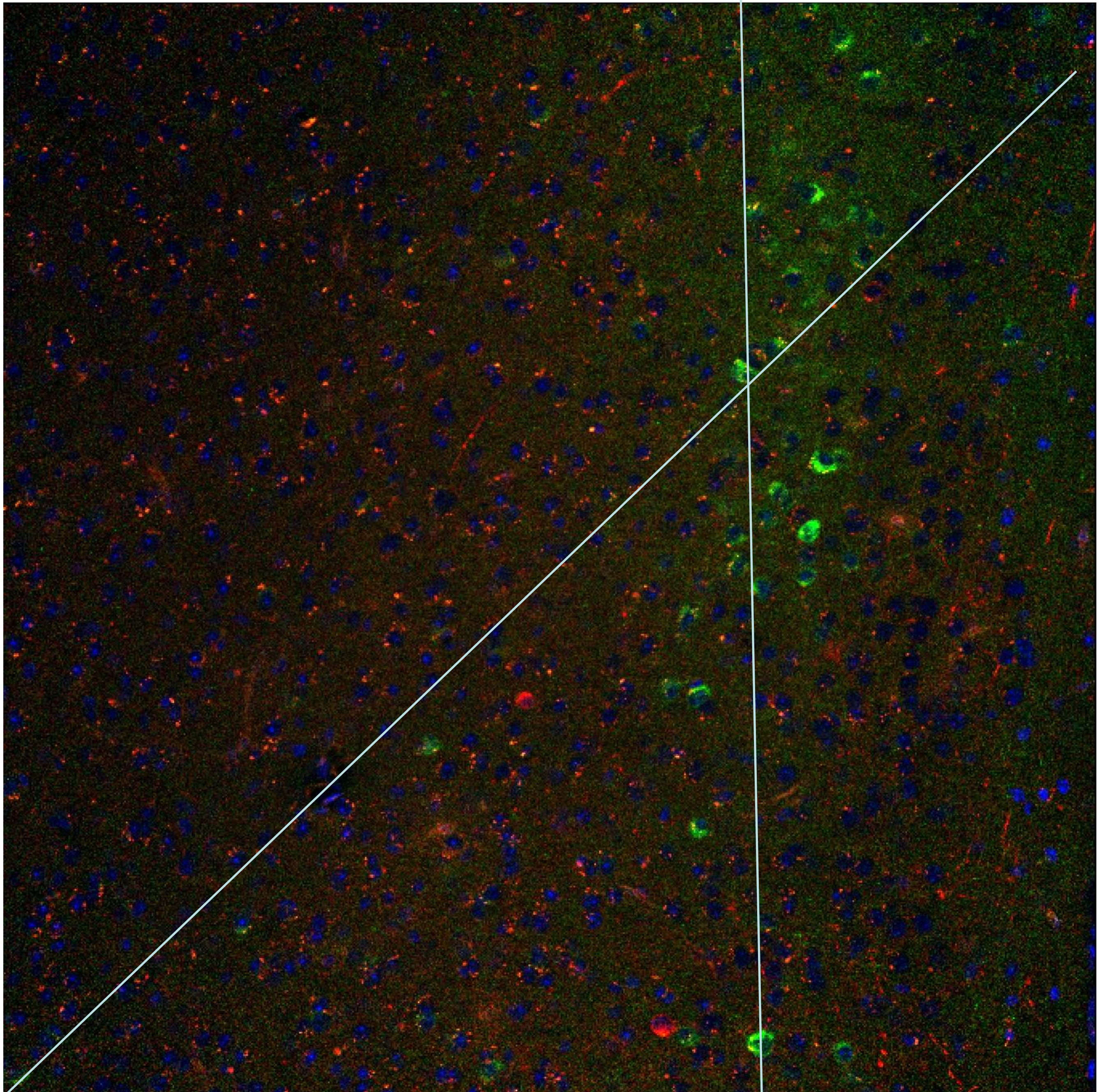

green：
Cg1 superficial：6
Cg1 deep：2
Cg2 superficial：7
Cg2 deep：9

red：
Cg1 superficial：0
Cg1 deep：1
Cg2 superficial：2
Cg2 deep：3

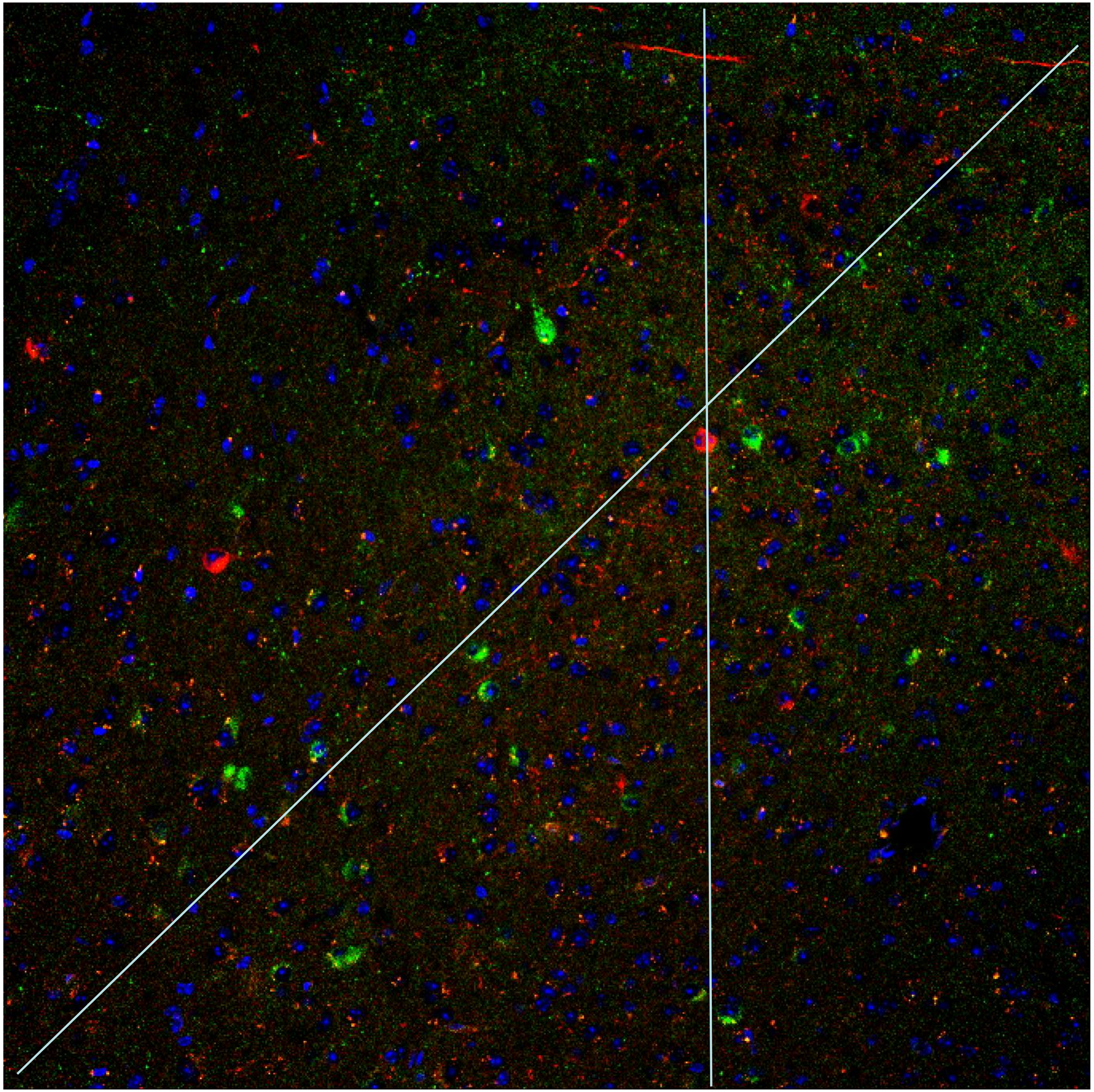

green：
Cg1 superficial：1
Cg1 deep：5
Cg2 superficial：7
Cg2 deep：7

red：
Cg1 superficial：3
Cg1 deep：3
Cg2 superficial：2
Cg2 deep：2

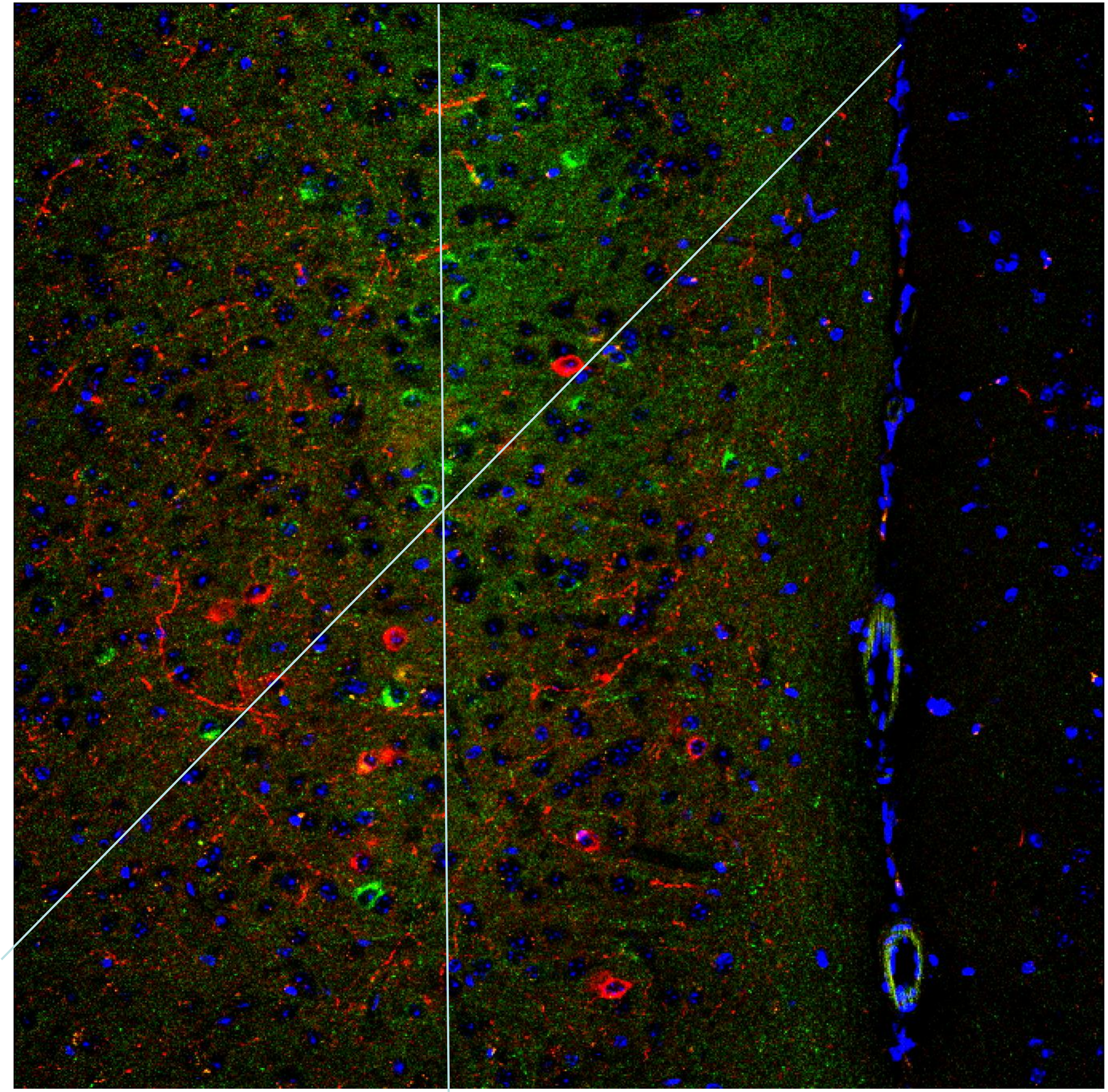

green：
Cg1 superficial：6
Cg1 deep：6
Cg2 superficial：3
Cg2 deep：2

red：
Cg1 superficial：1
Cg1 deep：2
Cg2 superficial：3
Cg2 deep：3

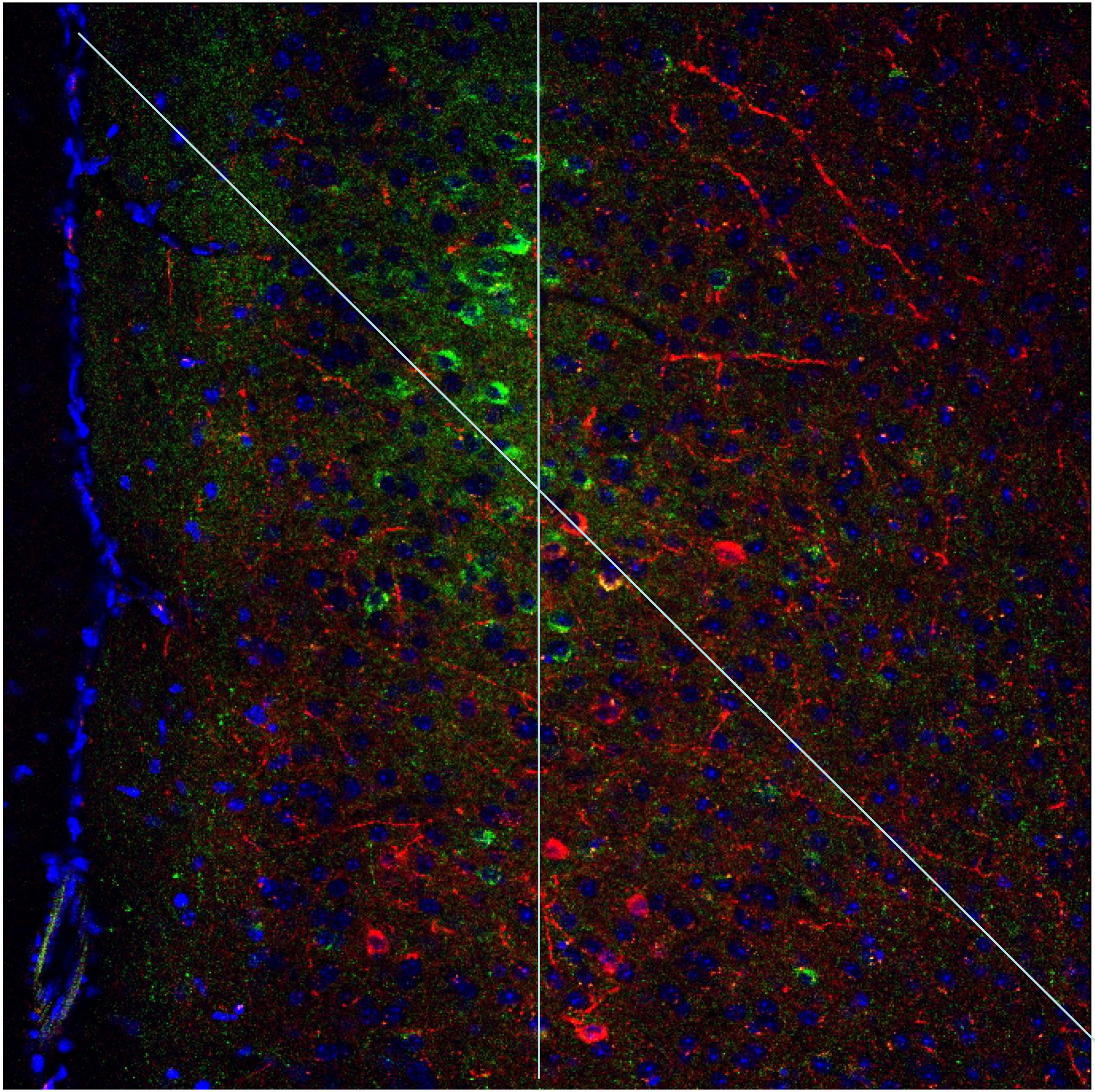

green：
Cg1 superficial：8
Cg1 deep：3
Cg2 superficial：9
Cg2 deep：4

red：
Cg1 superficial：0
Cg1 deep：2
Cg2 superficial：3
Cg2 deep：6

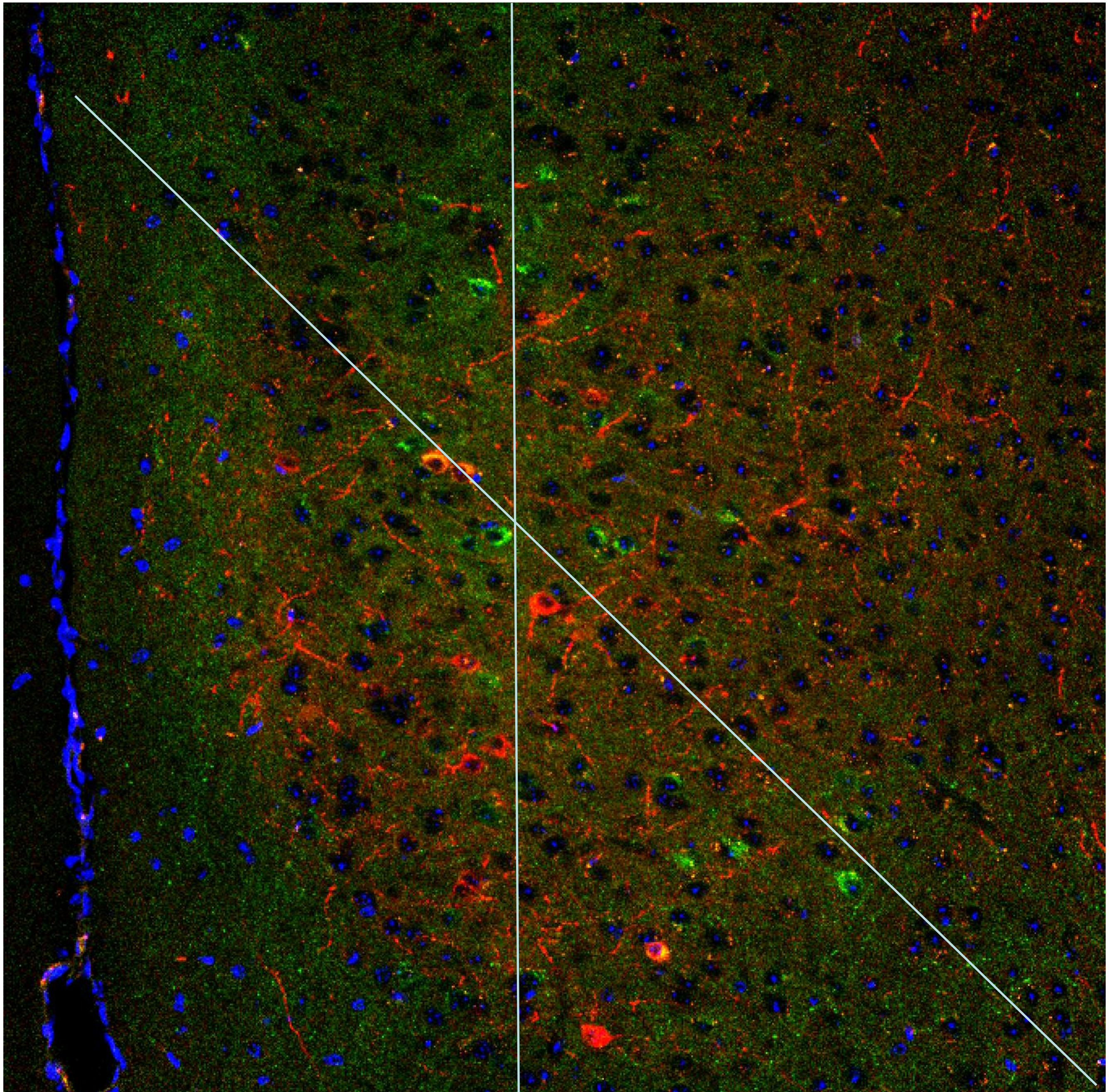

green：
Cg1 superficial：1
Cg1 deep：5
Cg2 superficial：2
Cg2 deep：3

red：
Cg1 superficial：1
Cg1 deep：2
Cg2 superficial：5
Cg2 deep：3

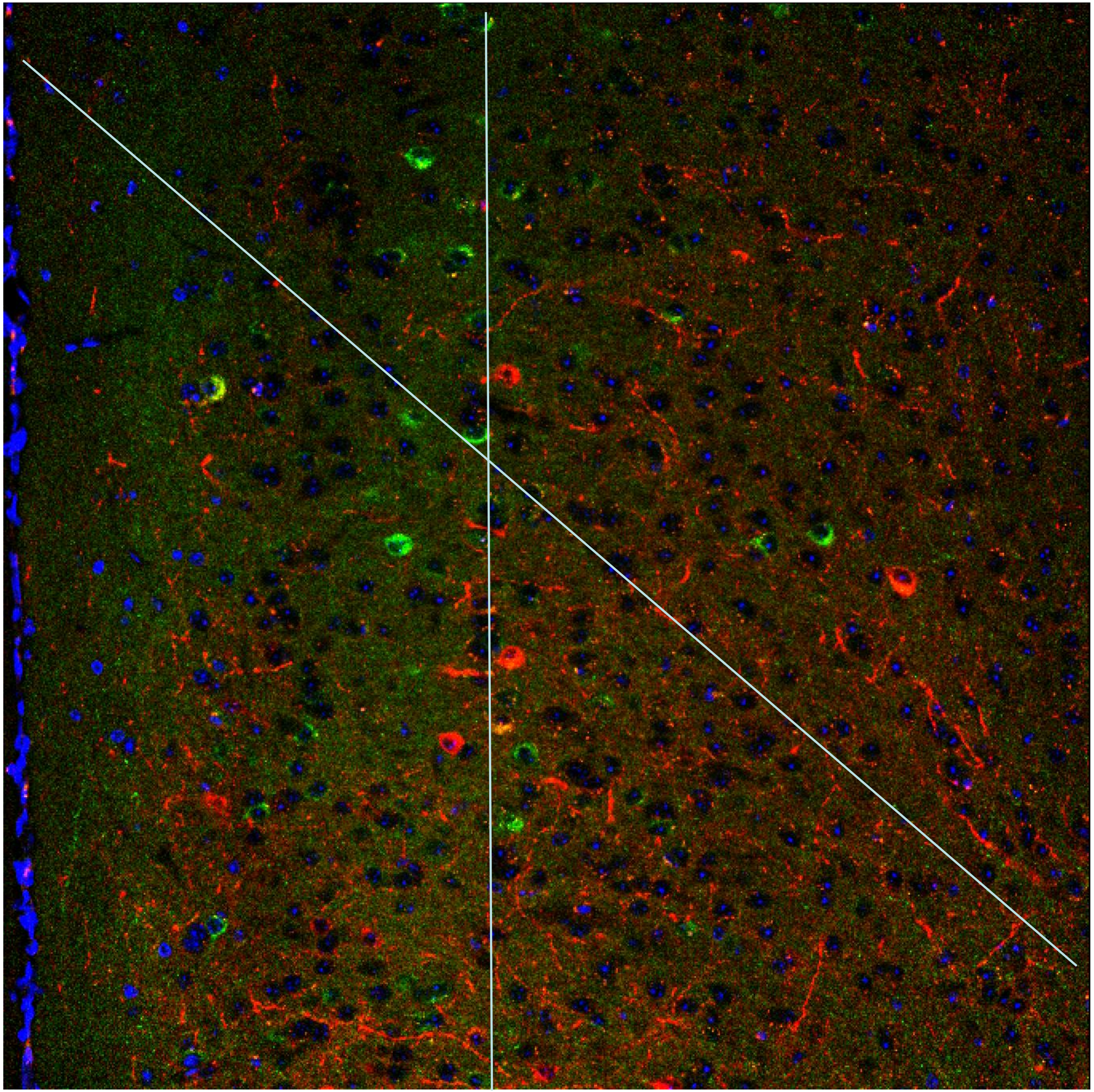

green：
Cg1 superficial：6
Cg1 deep：5
Cg2 superficial：6
Cg2 deep：3

red：
Cg1 superficial：0
Cg1 deep：3
Cg2 superficial：4
Cg2 deep：2

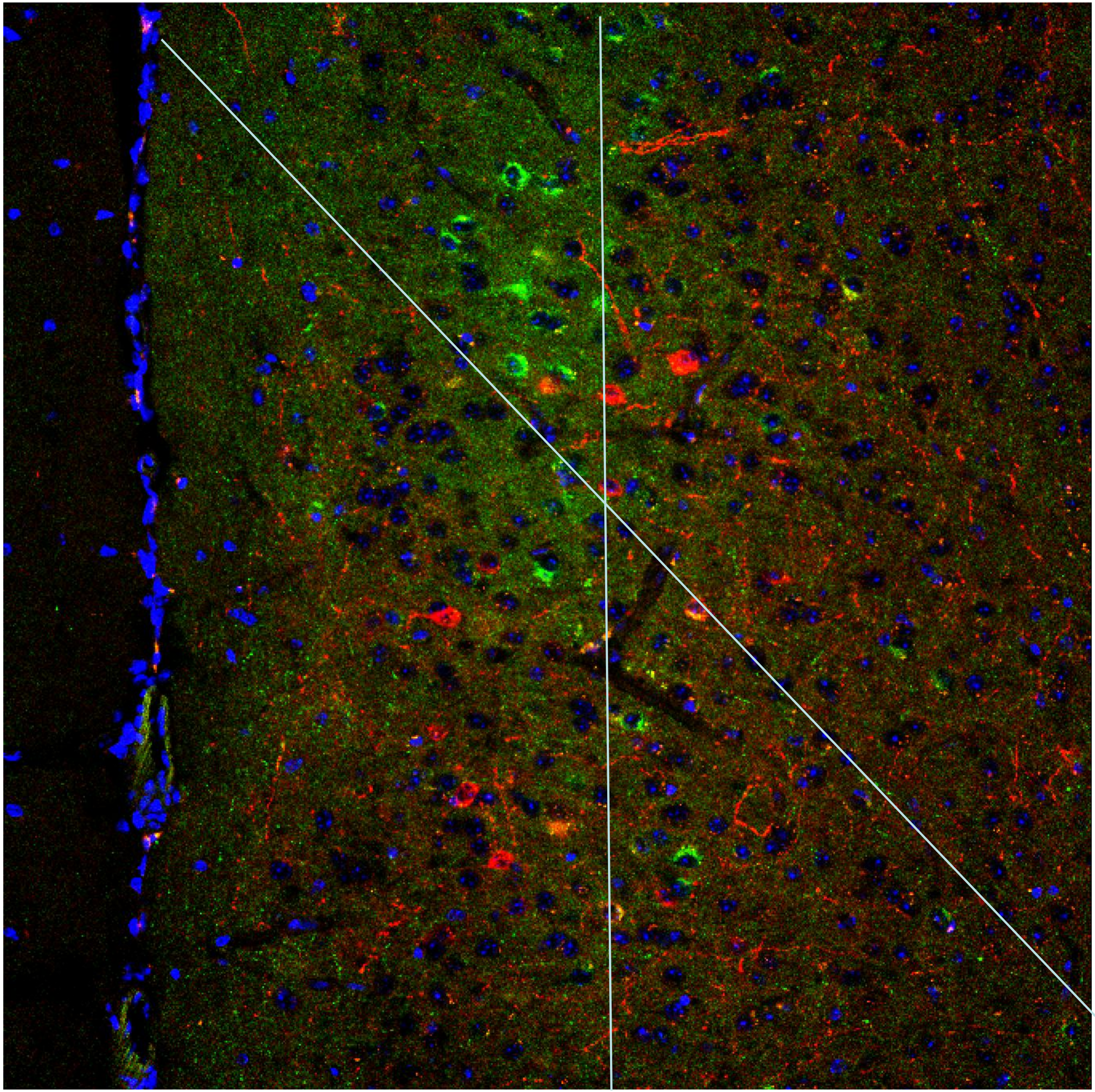

green：
Cg1 superficial：7
Cg1 deep：4
Cg2 superficial：3
Cg2 deep：3

red：
Cg1 superficial：1
Cg1 deep：4
Cg2 superficial：4
Cg2 deep：2

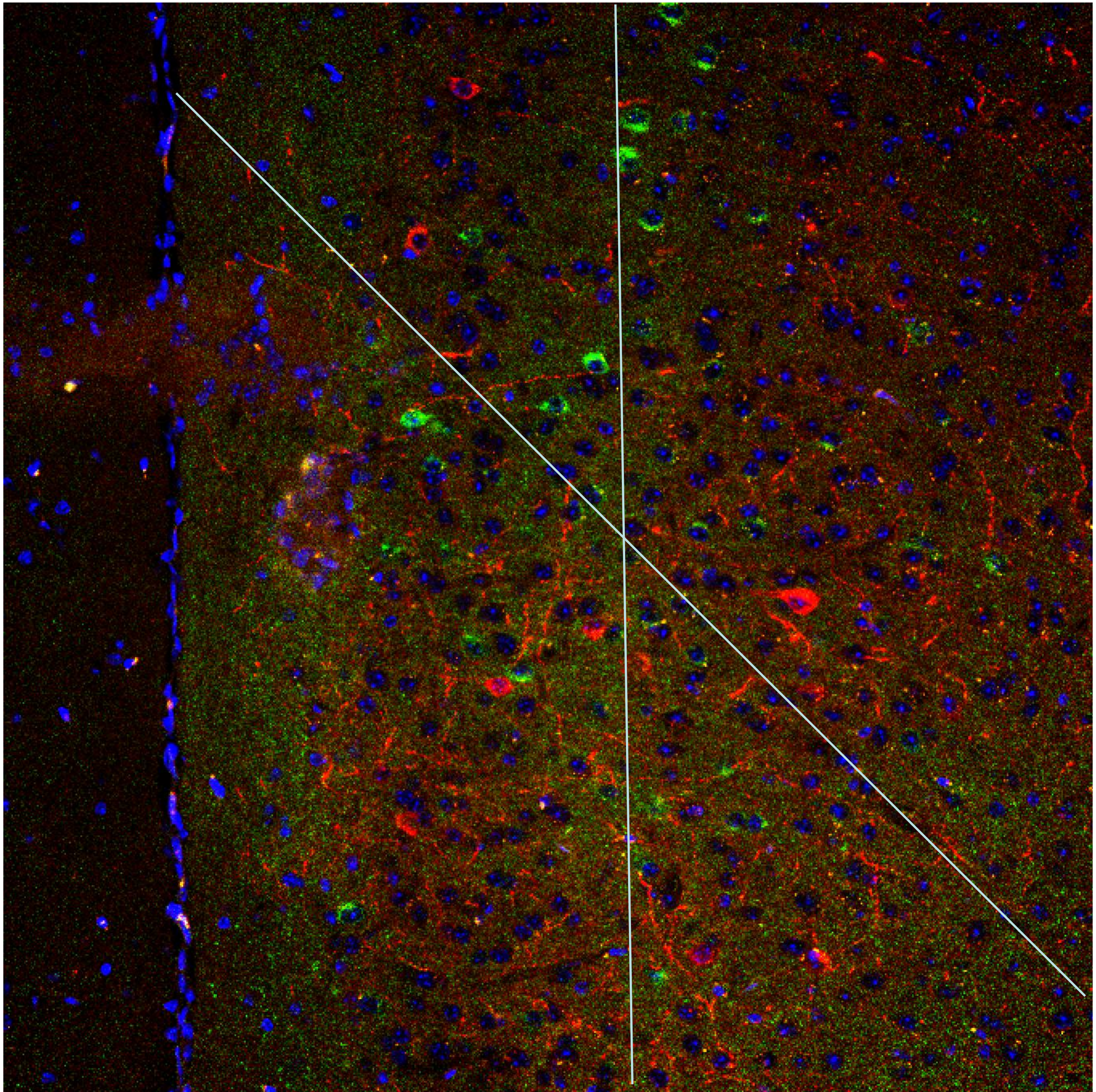

green：
Cg1 superficial：4
Cg1 deep：6
Cg2 superficial：6
Cg2 deep：2

red：
Cg1 superficial：2
Cg1 deep：5
Cg2 superficial：4
Cg2 deep：3
