# Supplementary material for: Distinct CaMKII neurons in anterior cingulate cortex regulate anxiety-like behavior and appetite suppression in a model of inflammatory pain
Source: Front Pharmacol. 2026 Sep 3;17:1897103. doi: 10.3389/fphar.2026.1897103 (PMC13603023; doi:10.3389/fphar.2026.1897103)
Supplement: Supplementary file 2 [file DataSheet2.pdf]

Table 1. Counts of ACC neurons projecting to BLA or NAc, stratified by cg subregion and cortical layer

Section ID	BLA-projecting neurons (cg1 superficial)	BLA-projecting neurons (cg1 deep)	BLA-projecting neurons (cg2 superficial)	BLA-projecting neurons (cg2 deep)	NAc-projecting neurons (cg1 superficial)	NAc-projecting neurons (cg1 deep)	NAc-projecting neurons (cg2 superficial)	NAc-projecting neurons (cg2 deep)
1	24	34	62	18	1	37	7	16
2	5	5	11	9	0	4	6	3
4	0	11	5	6	1	5	3	3
5	7	11	4	3	1	2	4	3
6	8	3	5	0	4	4	6	8
7	12	11	5	3	2	2	6	5
8	6	2	7	9	0	1	2	3
9	1	5	7	7	3	3	2	2
10	6	6	3	2	1	2	3	3
11	8	3	9	4	0	2	3	6
12	1	5	2	3	1	2	5	3
13	6	5	6	3	0	3	4	2
14	7	4	3	3	1	4	4	2
16	4	6	6	2	2	5	4	3
